# Tailoring Regioselectivity‐Controlled UDP‐Glycosyltransferase for Bidirectional Glycosylation of Tyrosol via Free Energy‐Driven Pocket Reshaping and Tunnel Engineering

**DOI:** 10.1002/advs.202509814

**Published:** 2025-08-04

**Authors:** Ziyu Zhang, Jingyi Chen, Zuozhi Liang, Wenming Shao, Zhen Gao, Bin Wu, Bingfang He, Gerhard Schenk

**Affiliations:** ^1^ College of Biotechnology and Pharmaceutical Engineering Nanjing Tech University 30 Puzhunan Road Nanjing 211816 China; ^2^ School of Pharmaceutical Sciences Nanjing Tech University 30 Puzhunan Road Nanjing 211816 China; ^3^ School of Chemistry and Molecular Biosciences The University of Queensland Brisbane QLD 4072 Australia

**Keywords:** free‐energy landscape, icariside D2, protein engineering, salidroside, UDP‐Glycosyltransferase

## Abstract

UDP‐glycosyltransferases (UGTs) are pivotal biocatalysts for synthesizing pharmaceutically valuable active components; however, their application is frequently constrained by poor regioselectivity and suboptimal catalytic efficiency. In this study, a tailored, free energy‐driven, substrate‐binding pocket reshaping strategy is implemented to pinpoint the specific residues in UGT_BL_1 that control bidirectional regioselective glycosylation of tyrosol, enabling the synthesis of salidroside and icariside D2 without the need for large‐scale screening. Additionally, modifications in the tunnel lead to two strictly regioselective mutants with improved catalytic efficiency due to the faster release of the products. Remarkably, while wild‐type UGT_BL_1 exhibits poor regioselectivity toward the alcoholic and phenolic hydroxyl groups of tyrosol, generating an almost equal mixture of products (1:1 ratio), mutant M2 achieved 99.2% regioselectivity toward the alcoholic hydroxyl group of tyrosol, coupled with a 14.8‐fold enhancement in catalytic efficiency for salidroside production. Similarly, mutant M2‐1 displays 99.1% regioselectivity toward the phenolic hydroxyl group, along with a 3.6‐fold improvement in catalytic efficiency for icariside D2 synthesis. Molecular dynamics simulations reveal details about the mechanism for improved regioselectivity and catalytic efficiency. This work provides important insights for protein engineering of UDP‐glycosyltransferase with the spacious active pocket in constructing small but smart mutant libraries.

## Graphical abstract

To address inherent limitations in regioselectivity and catalytic efficiency of UDP‐glycosyltransferases, an integrated strategy combining free‐energy driven pocket reshaping and tunnel engineering is developed. This strategy enables UGT_BL_1 to bidirectional glycosylation of tyrosol, synthesizing salidroside or icariside D2 without extensive screening experiments.This study provides key insights to construct small but smart mutant library for  enzyme engineering.

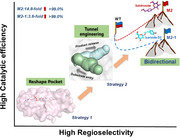

## 1 Introduction

Glycosides derived from medicinal herbs have attracted considerable attention owing to their diverse pharmacological activities, and are currently used globally as therapeutic drugs, food additives, and nutraceuticals in the healthcare industry.^[^ ^1^, ^2^ ^]^ Salidroside, the 8‐O‐β‐D‐glucoside derivative of tyrosol, is the main bioactive ingredient of Rhodiola plants, which not only has neuroprotective, cardioprotective, and hepatoprotective effects, but also exhibits anti‐hypoxia, anti‐aging, and anti‐fatigue activities.^[^ ^3^, ^4^ ^]^ Icariside D2, the 4‐O‐β‐D‐glucoside derivative of tyrosol, and a regio‐isomer of salidroside, is mainly isolated from the plants Epimedium and Rhodiola.^[^ ^5^, ^6^ ^]^ It has been shown to have remarkable anticancer activity against leukemia cells in vitro.^[^ ^7^, ^8^ ^]^ Currently, direct extraction from the plants, chemical synthesis, and microbial biosynthesis have been developed to produce salidroside and icariside D2.^[^ ^9^, ^10^, ^11^, ^12^ ^]^ Among them, microbial biosynthesis has the greatest promise to meet commercial demand sustainably due to its environmental friendliness, low energy consumption, high production efficiency and simplicity of operation. To date, *Escherichia coli* and *Saccharomyces cerevisiae* stains strains have been engineered for the de novo synthesis of salidroside and icariside D2. In the corresponding biosynthetic pathway, the last step involves glycosylation of tyrosol. Specifically, tyrosol undergoes glycosylation at the alcoholic or phenolic positions, resulting in the formation of salidroside or icariside D2, respectively.^[^ ^12^, ^13^, ^14^ ^]^

Glycosyltransferases (EC 2.4.x.y) constitute an enzyme superfamily that transfer activated glycosyl moieties to an acceptor substrate. Among them, enzymes belonging to Family 1, often referred to as uridine diphosphate glycosyltransferases (UGTs), are mainly responsible for the glycosylation of natural products in plants.^[^ ^15^ ^]^ To date, several plant UGTs have been utilized to synthesize salidroside or icariside D2.^[^ ^16^, ^17^ ^]^ However, the majority of UGTs display substantial product promiscuity, leading to poor regioselectivity. Only three plant UGTs have exhibited strict regioselectivity in synthesizing salidroside and icariside D2, i.e., *At*UGT85A1 from *Arabidopsis thaliana*, which specifically synthesizes salidroside,^[^ ^18^, ^19^ ^]^ and *Rc*UGT1 from *Rhodiola crenulata* and *Rr*UGT3 from *Rhodiola rosea*, both of which exclusively generate icariside D2.^[^ ^19^, ^20^ ^]^ However, low enzymatic activity and poor expression efficiency have limited their applications. Although microbial‐derived UGTs exhibit higher expression efficiency and greater enzymatic activity, their more spacious active pockets lead to poor regioselectivity.^[^ ^21^, ^22^ ^]^ For example, glycosyltransferase YjiC from *Bacillus subtilis* has been shown to glycosylate tyrosol at both the phenolic and alcoholic positions, producing salidroside and icariside D2 in an approximately 1:1 ratio.^[^ ^23^ ^]^ The glycosylation of resveratrol, catalyzed by glycosyltransferase YjiC from *B. licheniformis* DSM 13, generates complex mixtures containing four glucoside derivatives, i.e., 3‐O‐, 4′‐O‐, 3,5‐O‐, and 3,5,4′‐O‐β‐glucoside.^[^ ^24^ ^]^ The concurrent formation of these regio‐isomers complicates downstream purification and hinders obtaining each compound in pure form for commercial applications. Therefore, the implementation of regio‐control in microbial UGTs is an important challenge to establish industrially relevant biosynthetic pathways for salidroside or icariside D2.

Protein engineering has emerged as a powerful tool to improve regioselectivity of enzymes.^[^ ^25^, ^26^ ^]^ For example, the UGT MiCGT from *Mangifera indica* was evolved using alanine scanning combined with iterative saturation mutagenesis. From that study a quadruple mutant was obtained that displayed strict 3‐O glycosylation selectivity toward quercetin.^[^ ^27^ ^]^ Similarly, directed evolution of UGT_BS_ from *Bacillus subtilis* 168 by modifying the residues around the active center improved 3‐OH glycosylation significantly,^[^ ^28^ ^]^ while the mutability landscape of hotspots residues in UGT YjiC from *B. licheniformis* was employed to design mutants with enhanced regioselectivity.^[^ ^23^ ^]^ These studies have demonstrated that the application of structure‐based rational and semi‐rational engineering was essential for obtaining a small but smart mutation library aimed at increasing regioselectivity of UGTs. Nevertheless, remarkably enhancing the regioselectivity of UGTs remains a challenge, probably attributed to the insufficient availability of molecular templates for robust modeling, as well as a limited understanding of the underlying reaction mechanisms.

**Figure 1:**
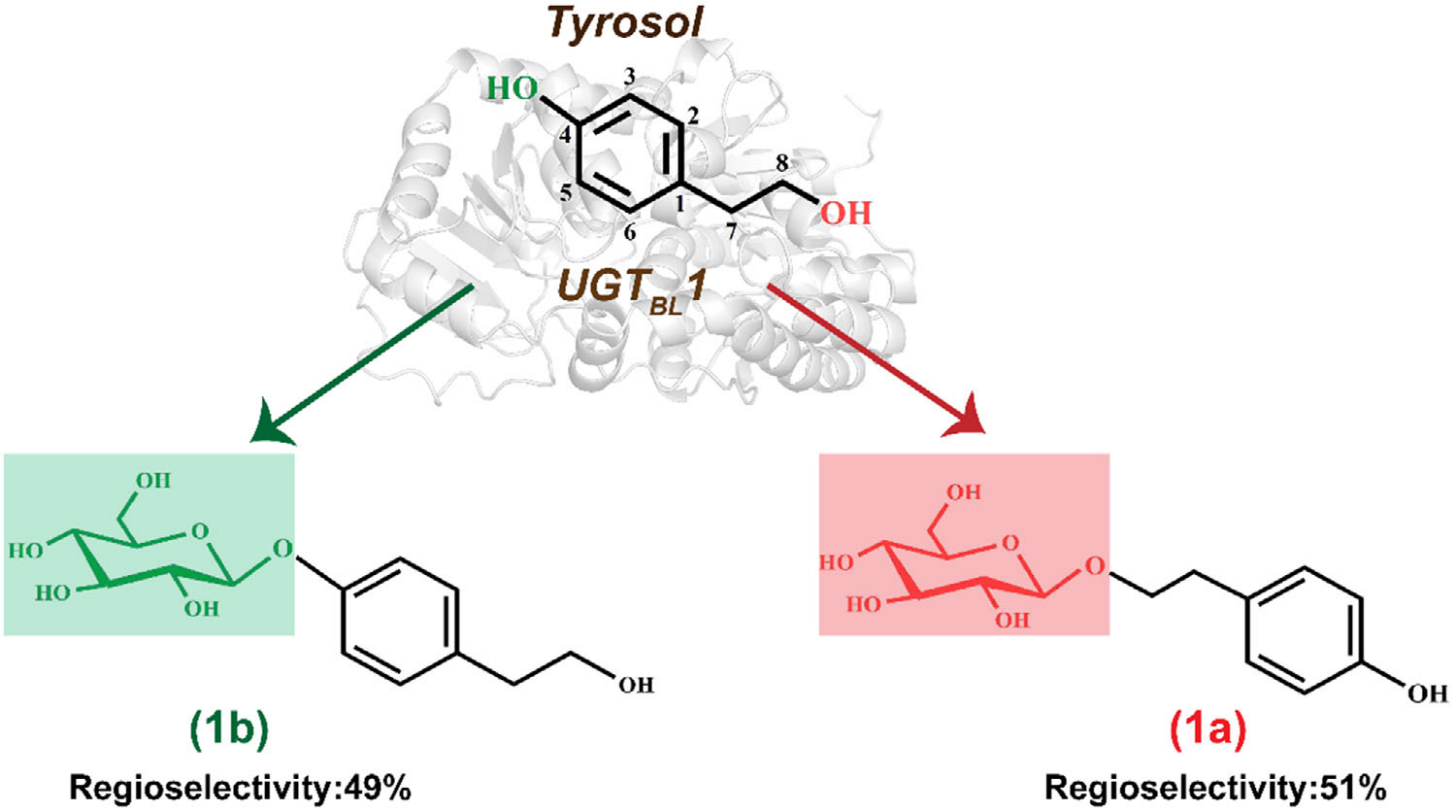
Scheme of the synthesis of salidroside (1a) and icariside D2 (1b) from tyrosol catalyzed by the glycosyltransferase UGT_BL_1.

In our previous study, a UGT (UGT_BL_1) from *Bacillus licheniformis*, capable of glycosylating tyrosol, was mined through the alignment and evolution of key motifs of the targeted enzyme UGT73B6 from *Rhodiola*.^[^ ^29^ ^]^ Compared to plant‐derived UGTs, UGT_BL_1 demonstrates good soluble expression in *E.coli* BL21(DE3). However, wild‐type UGT_BL_1 has poor regioselectivity glycosylating the alcoholic hydroxyl (C8‐OH) and phenolic hydroxyl (C4‐OH) groups in tyrosol with similar efficiency, resulting in the production of salidroside and icariside D2 in an approximately 1:1 ratio (**Figure** **1**). In this work, we successfully engineered UGT_BL_1 mutants capable of efficiently producing salidroside and icariside D2 with significantly enhanced catalytic performance and regioselectivity. Our approach was based on an initial computational analysis to predict the preferred binding orientation of tyrosol within two adjacent cavities in the binding pocket of UGT_BL_1. Based on the principal component analysis of the free energy landscape of the protein‐ligand complex in the spacious pocket, we constructed a small but smart mutant library designed to enable the bidirectional regulation of the regioselectivity of UGT_BL_1. To further improve the catalytic efficiency of the engineered mutants, site‐directed saturation mutagenesis and combinatorial mutagenesis on residues lining the substrate entry and product release tunnels were performed. Finally, molecular dynamics (MD) simulations were conducted to illustrate the molecular‐level mechanisms underlying the enhanced regioselectivity and catalytic efficiency of the engineered mutants.

## 2 Results and Discussion

### 2.1 Analysis of Substrate‐Binding Patterns and Prediction of Key Residues Responsible for Bidirectional Regioselective Control

**Figure 2:**
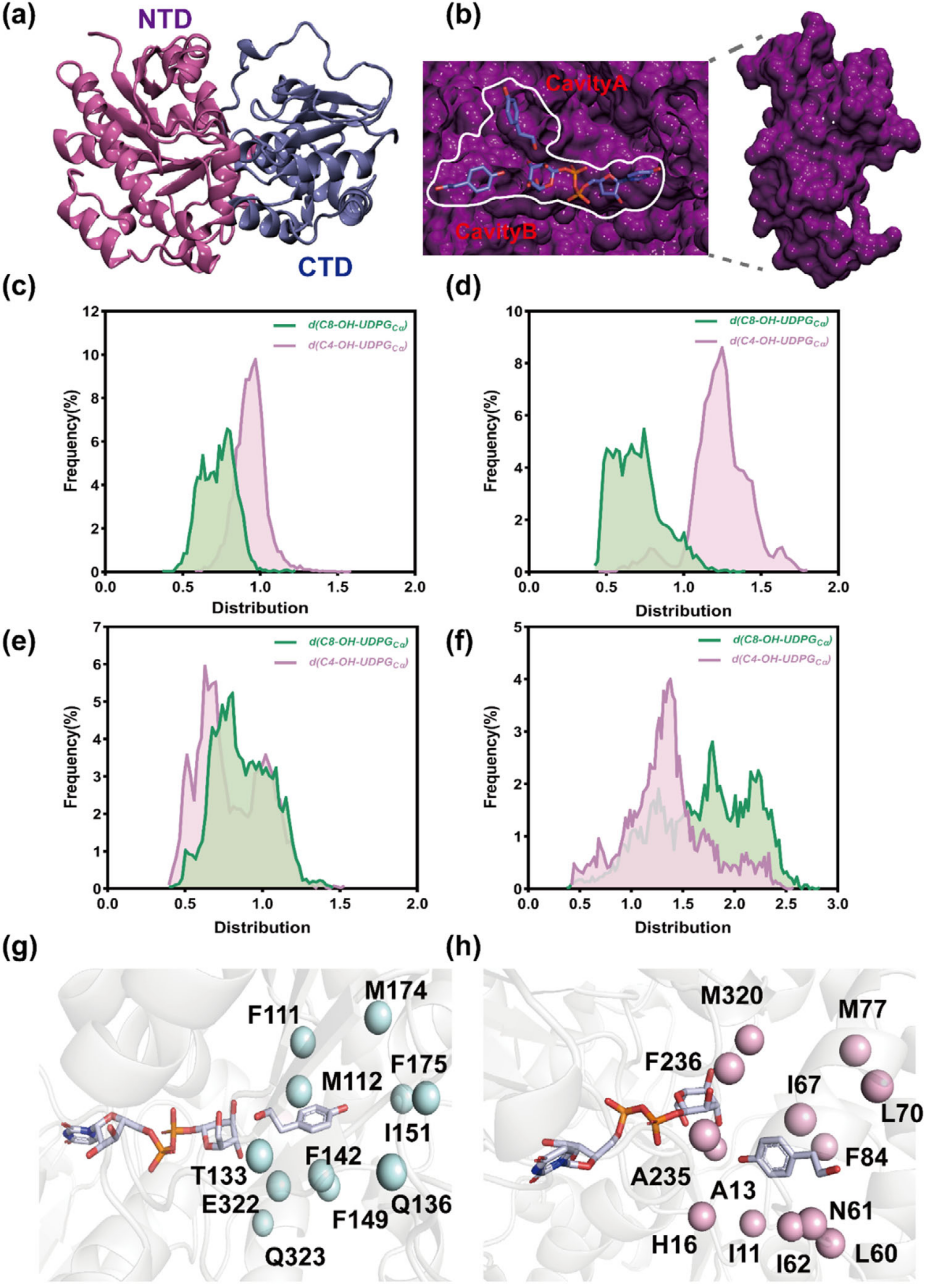
Structural determination of UGT_BL_1. a) The structural model of UGT_BL_1 has a GT‐B overall fold, consisting of two Rossman‐like domains in the N (purple) and C‐terminus (blue). b) The docking of ligands (UDPG and tyrosol) in cavities A and B of UGT_BL_1, and an enlarged view of the pocket, shown as a dark purple surface. c–f) Frequency distribution of the distances between the C4‐OH and C8‐OH of tyrosol and the Cα of UDPG in the complexes A/C4‐OH, A/C8‐OH, B/C4‐OH and B/C8‐OH, respectively. g,h) Amino acid residues within a 5 Å radius of tyrosol in the pocket of wild‐type UGT_BL_1. UDPG and tyrosol are shown as sticks and identified residues are represented in pale cyan spheres for cavity A (a) and pink spheres for cavity B (b).

UGT_BL_1 catalyzes the glycosylation of phenolic aglycones to produce glycosides such as salidroside and icariside D2. However, poor regioselectivity combined with insufficient understanding of the catalytic mechanism limited the application of UGT_BL_1.^[^ ^29^ ^]^ To gain insight to the structural basis for the substrate promiscuity of UGT_BL_1, molecular docking and molecular dynamic simulations were performed. A 3D model of UGT_BL_1 was generated using the AlphaFold 2 server. The overall structure adopts the conserved GT‐B fold, similar to other GT1 UGTs, with two β/α/β Rossmann‐fold domains separated by a large cleft (**Figure** **2**a).^[^ ^15^ ^]^ Subsequently, structural model for UGT_BL_1 complex with the donor UDPG and the acceptor tyrosol was generated through structural alignment to the homologous complex structure UGT109A1 from *B*. *spizizenii* (PDB ID: 7VLB). Interestingly, we found that UGT_BL_1 contains two potential binding cavities (Cavity A and B) close to the catalytic center, as well as the UPDG binding site (Figure 2b). A highly conserved histidine residue (H16), common among O‐glycosyltransferases, was hypothesized to act as the catalytic base for the deprotonation of hydroxyl group. The generated nucleophile then attacks the Cα of the glucose moiety in UDPG, using an S_N_2‐like mechanism, where residues H16 and D110 together form the catalytic dyad (Figure S1, Supporting Information), supporting by the observation that alanine substitution of either H16 or D110 completely abolished glycosylation activity.^[^ ^30^, ^31^ ^]^ Close examination of the docking results in cavity B, we observed that the docked tyrosol, possessing both phenolic and alcoholic hydroxyl groups are close to H16, thus capable of undergoing glycosylation to produce either salidroside or icariside D2. A similar phenomenon is also observed when tyrosol was docked into cavity A of UGT_BL_1 (Figure S3, Supporting Information).

To assess the binding preference of tyrosol in the two cavities, we calculated the docking free energy of this substrate in each cavity with two orientations. In cavity A, the binding free energies of tyrosol in the binding orientations favoring the generation of salidroside (designated as A/C8‐OH) and the icariside D2 (designated as A/C4‐OH) are −59.51 kcal mol^−1^ and −55.38 kcal mol^−1^, respectively. Similarly, in cavity B, the binding energy are calculated as −63.11 kcal mol^−1^ for the conformation favoring the formation of salidroside (designated as B/C8‐OH) and −60.43 kcal mol^−1^ for that leading to icariside D2 (designated as B/C4‐OH) (Figure S4, Supporting Information). These calculations indicate that UGT_BL_1 might exhibit no clear preference for glycosylation of tyrosol at either the C8‐OH or C4‐OH position, as both cavities display comparable binding free energies. To further evaluate the binding states of tyrosol in the two cavities, four representative conformations were subjected to 100 ns of MD simulation (Figure S5, Supporting Information). Interestingly, the measurements of the distance between hydroxyl group of tyrosol and the Cα of UDPG in two cavities were drastically different after the 100 ns simulations. In cavity A, the C8‐OH of tyrosol is in close proximity to the Cα of UDPG, in contrast to the C4‐OH, which is located adjacent to the Cα of UDPG in cavity B (Figure 2c–f). These results suggest that the cavity A appears to promote the generation of salidroside, while cavity B seems to facilitate the production of icariside D2. Collectively, our observations of separate favored dual‐conformations in the two binding cavities offers a plausible explanation for the ability of UGT_BL_1 to produce salidroside and icariside D2 with an approximately 1:1 ratio. As a consequence, these structure insights guided further enzyme engineering efforts aimed to improve the bidirectional regioselectivity of UGT_BL_1.

**Figure 3:**
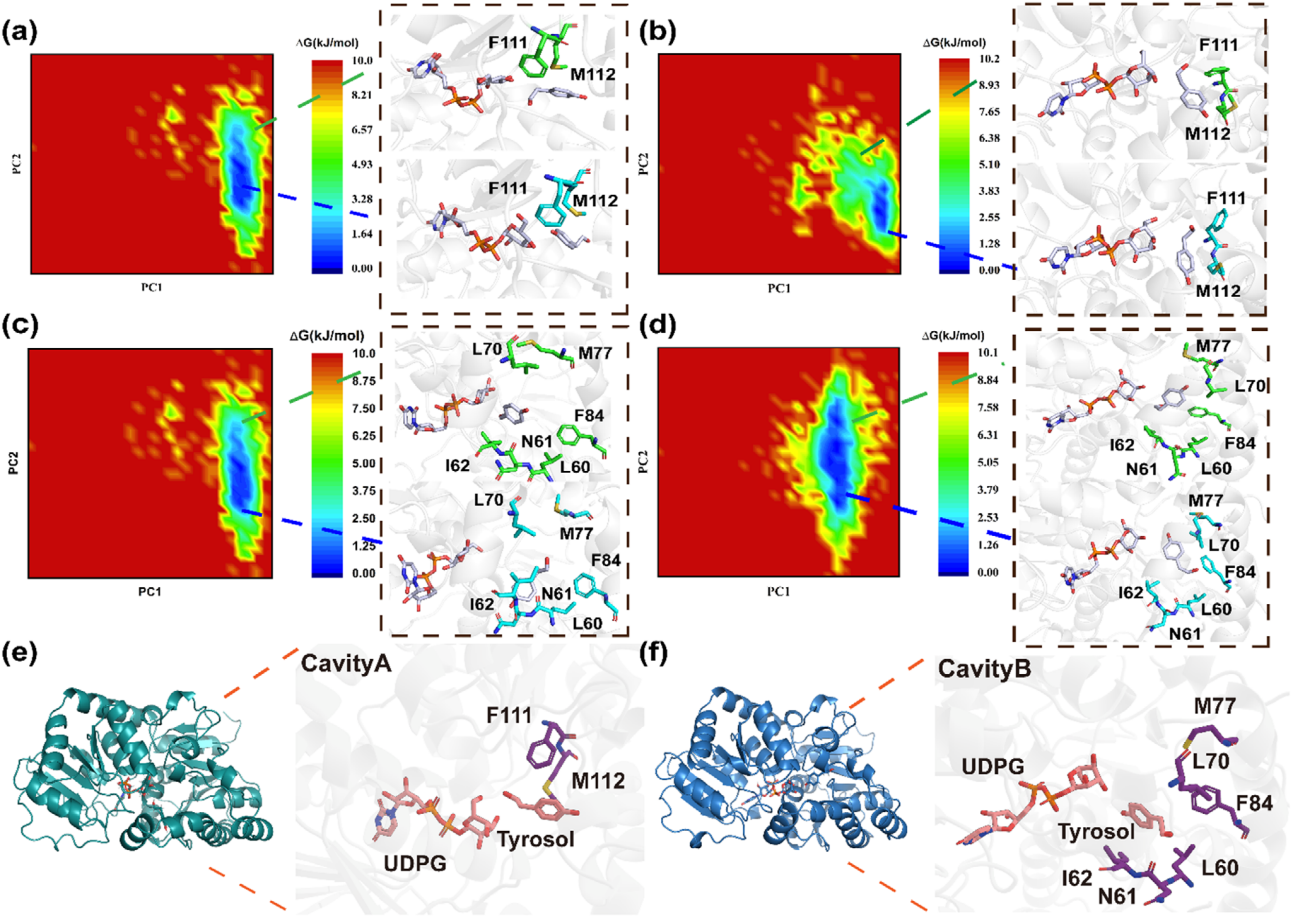
The free energy landscape and free energies of representative states in the tyrosol binding process obtained from 100 ns MD simulations of the ternary complexes UGT_BL_1‐UDPG‐Tyrosol in cavities A and B. a) Free energy profile of tyrosol in the C8‐OH conformation and the key amino acid residues surrounding tyrosol in cavity A. b) Free energy profile of tyrosol in the C4‐OH conformation, along with key residues that surround it in cavity A. (c) and (d) represent the same states for tyrosol in cavity B as described in (a) and (b), respectively. e–f) Hotspot amino acids surrounding tyrosol in cavities A and B. Key residues surrounding the binding pocket and ligands are shown as sticks.

The substrate‐binding pocket has a significant influence on enzyme regioselectivity and catalytic efficiency by modulating the binding conformation and interactions of the substrate. To identify key residues regulating the regioselectivity of UGT_BL_1, we focused on the residues lining the two distinct cavities (A and B) within the binding pocket. A total of 11 residues in cavity A (F111, M112, T133, Q136, F142, F149, I151, M174, F175, E322, and Q323) and 13 residues in cavity B (I11, A13, H16, L60, N61, I62, I67, L70, M77, F84, A235, F236, and M320) were identified (Figure 2g,h). Given the large number of target residues, the construction and screening of the saturated mutant library posed a significant experimental challenge. We integrated principal component analysis (PCA) with free energy landscape (FEL) calculations to systematically evaluate four protein‐substrate complexes. Equilibrated molecular dynamics trajectories were analyzed by constructing 2D FELs using Cα backbone root‐mean‐square deviation (RMSD) and radii of gyration (Rg) as reaction coordinates, with the color scale representing the relative free energy (ΔG). FEL analysis pinpointed the global minimum corresponding to the most stable binding conformation and local minima denoting potential metastable states (**Figure** **3**a–d). Structural comparisons between these stable and metastable states revealed conserved interaction residues in both energy wells, particularly stabilizing hydrogen bonds and hydrophobic interactions. In cavity A, residues F111 and M112 engage in hydrophobic interactions with tyrosol throughout the simulation trajectory, stabilizing its binding pose for salidroside synthesis. In cavity B, N61 forms a stable hydrogen bond with tyrosol, while L60, I62, L70, M77, and F84 forms hydrophobic interactions with tyrosol. These interactions collectively stabilize the catalytically productive conformation for icariside D2 synthesis. Based on this analysis, F111 and M112 in cavity A were identified as potential hotspots for improving the regioselectivity for icariside D2, while L60, N61, I62, L70, M77, and F84 in the cavity B were chosen as critical hotspots for enhancing the regioselectivity for salidroside (Figure 3e,f).

### 2.2 Site‐Saturation and Combinatorial Mutagenesis of Selected UGT_BL_1 Residues

**Figure 4:**
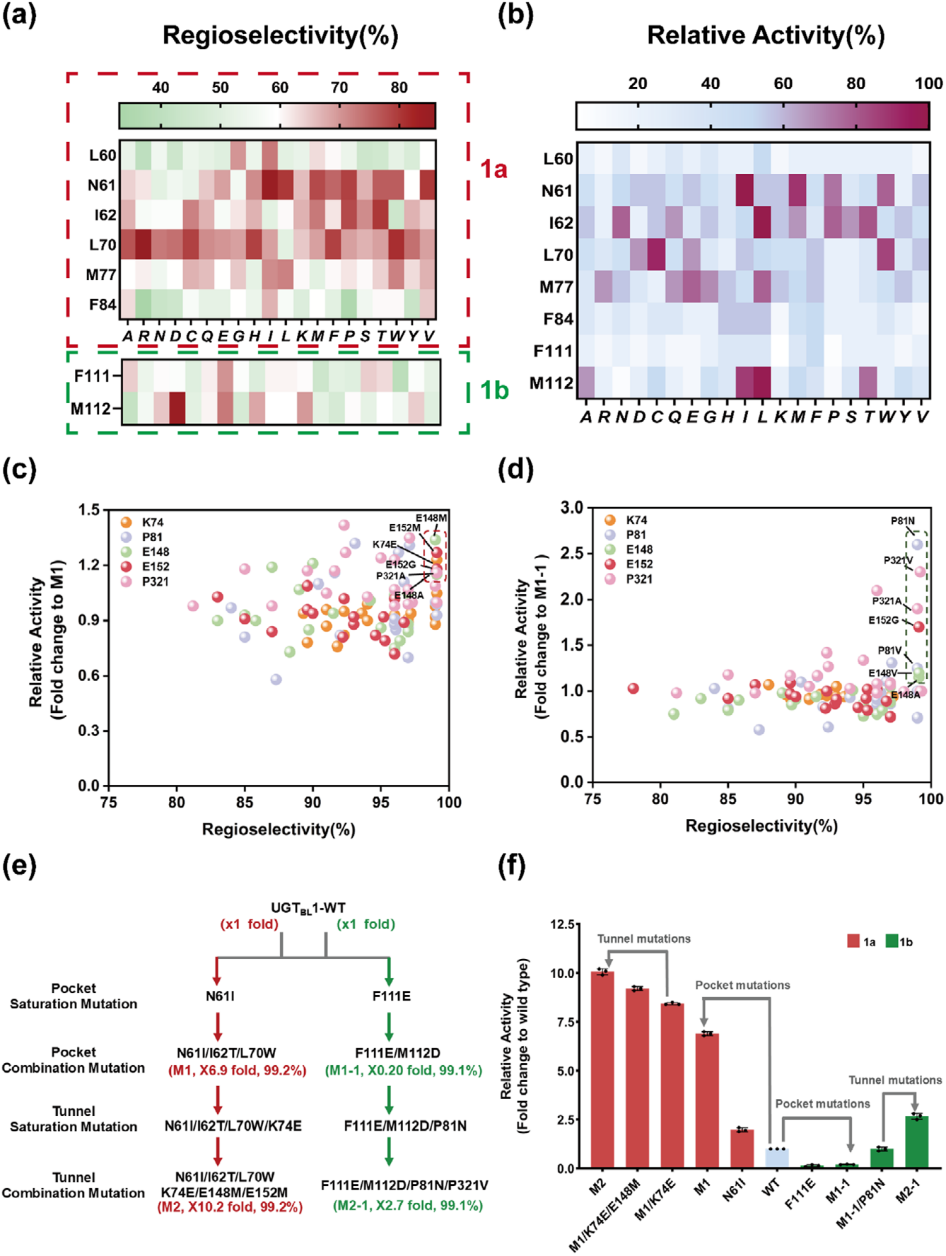
Design and screening of the mutagenesis library. a) Regioselectivity of mutational hotspots. b) Relative activities of UGT_BL_1 mutants to synthesize either salidroside or icariside D2. The relative activity was calculated using the highest enzyme activity as 100%. c,d) Relative activity and regioselectivity of UGT_BL_1 variants that have mutations in key residues within the product release tunnel, using either M1 and M1‐1 as templates, respectively. e) Design and evolution workflow used to generate mutants M2 and M2‐1. f) Combinational campaign for catalytic activity glycosylation mutants. Relative activity represents mean ± SD of three independent replicates (n = 3).

Single‐site saturated mutagenesis was performed on eight sites to achieve bidirectional control over the regioselectivity of UGT_BL_1 for tyrosol to synthesize salidroside or icariside D2. To improve regioselectivity in cavity B for salidroside synthesis, beneficial mutations were found at positions N61, I62, L70, and M77. For site N61, five mutants (N61I, N61L, N61M, N61P, and N61V) showed improved C8‐OH selectivity, with values ranging from 78% to 85%. Two favorable substitutions at site I62 were I62P and I62T, which exhibited the regioselectivity of 78% and 80% toward C8‐OH, respectively. At position L70, five mutants display improved the regioselectivity for C8‐OH, including L70R, L70C, L70H, L70F, and L70W. Among them, L70R possessed the highest regioselectivity (86%) for salidroside formation (**Figure** **4**a). At position 77, L77 and W77 displayed enhanced the regioselectivity of 77% and 75% to C8‐OH. Furthermore, mutants N61I, N61M, N61P, I62P, I62T, L70C, and L70W also have 1.5 to 2.0‐fold higher specific activity than wild‐type (Figure 4b). Our findings illustrate that even single mutation at key residue sites may improve both regioselectivity and catalytic activity of UGTs.

To improve regioselectivity of in cavity A for the production of icariside D2, screening identified beneficial mutations at both positions F111 and M112. As shown in Figure 4a,b, mutant F111E displays a strong preference for C4‐OH of tyrosol, resulting in an increase in the ratio of icariside D2 from 49% in the wild‐type enzyme to 80%. However, improved regioselectivity comes with a significant reduction in the specific activity by over 50% in comparison to the WT. Interestingly, at site M112, single substitutions displayed a diverse impact on regioselectivity; while mutant M112D exhibits improved C4‐OH selectivity (98.2%) but with decreased activity (25%), mutants M112I and M112L display elevated regioselectivity toward C8‐OH (≈85%) with a 1.5‐fold higher specific activity than that of WT.

To evaluate the feasibility of bidirectional control over the regioselectivity of UGT_BL_1 (i.e., in favor of either salidroside or icariside D2 synthesis), the GRAISM (greedy accumulated iterative site‐specific mutagenesis) strategy was performed on five residues. We targeted cavity B to engineer UGT_BL_1 for increased regioselectivity toward the C8‐OH group of tyrosol to produce salidroside. N61I was chosen as the initial template, and positions I62, L70 and M77 were individually subjected to iterative cycles. Specifically, double mutant N61I/I62T (96%) is the most effective candidate after the first round of mutagenesis with a 2.3‐fold improvement in catalytic activity relative to WT. It was subsequently used as template for mutagenesis at position. Among the resulting triple mutants, N61I/I62T/L70W (M1) led to further improvement of C8‐OH selectivity (99.2%). Moreover, the specific activity of this triple mutant is 3.0‐fold and 6.9‐fold higher than those of N61I/I62T double mutant and UGT_BL_1, respectively (Figure 4e,f). When L77 and W77 were incorporated into M1 to generate the quadruple mutants, no additional improvements in regioselectivity or catalytic activity were observed relative to M1.

As mentioned above, the two mutants M112L and M112I in cavity A also demonstrate high regioselectivity toward C8‐OH. However, when these mutations were introduced into M1 to generate two quadruple mutants, M1/M112L and M1/M112I, no further improvements in regioselectivity and catalytic activity relative to M1. Previous studies demonstrated the significance of the residue in position 62 and 112 in enhancing regioselectivity in UGT_BL_1; an alanine scan of 19 amino acid residues in the substrate pocket identified mutations those two positions as beneficial for enhanced production of resveratrol.^[^ ^32^ ^]^ Similarly, saturation mutagenesis using a mutational landscape analysis of amino acid residues in the substrate pocket of *Bl*YjiC also found that I62 and M112 had a substantial impact on the regioselectivity for tyrosol.^[^ ^23^ ^]^ Nonetheless, compared to previous studies required extensive experimental efforts, our study successfully pinpointed three residues with improved C8‐OH regioselectivity for tyrosol through mutations of only six hotspots, bypassing the need for laborious large‐scale screening.

We also focused attention on cavity A to engineer UGT_BL_1 variants with high regioselectivity toward the C4‐OH group of tyrosol to produce icariside D2. By generating and screening iterative mutagenesis libraries in positions 111 and 112, the double mutant F111E/M112D (M1‐1) was obtained with high C4‐OH selectivity (99.1%) and a specific activity of 20% of that of wild‐type UGT_BL_1 (Figure 4e,f). In previous studies aimed improving regioselectivity of glycosyltransferases, Zhu et al. employed the glycosyltransferase UGT_BS_ to synthesize resveratrol glucoside by mutating residues within 3.5 Å of the binding pocket, successfully generating mutants with enhanced regioselectivity toward the 3‐hydroxyl group.^[^ ^21^ ^]^ Similarly, Wen et al. used glycosyltransferase MiCGT to synthesize flavonoid compounds by mutating residues within 5 Å of quercetin, achieving mutants with strict regioselectivity for the 3‐hydroxyl group of quercetin.^[^ ^27^ ^]^ In contrast, this study adopted a free energy‐driven pocket remodeling strategy, pinpointing only two residues within the binding pocket, ultimately obtaining mutant M1‐1, which is capable of directed synthesis of icariside D2 with a regioselectivity of 99.1%. Thus, our “dual‐conformation” binding mechanism and free energy‐driven pocket reshaping strategy enables the rational engineering of the expansive substrate binding pocket of UGT_BL_1, allowing for bidirectional regulation of tyrosol regioselectivity without requiring extensive large‐scale screening.

The precise identification of hotspots is important for constructing mutation libraries that maximize the probability of achieving ideal results while minimizing experimental effort, thereby creating “small but smart” libraries. The free‐energy driven pocket reshaping strategy reduced the number of target residues within the binding pocket from 23 to 8, significantly reducing experimental workload. Single‐site saturation mutagenesis (SSM) was then performed on these 8 candidate residues using the redundancy‐reducing degenerate primer NNK (where N = A/C/G/T and K = G/T, encoding all 20 amino acids). To achieve >95% coverage of all possible amino acid substitutions at a specific site, it was theoretically necessary to screen ≈94 transformants per residue.^[^ ^33^, ^34^ ^]^ Consequently, a total of 752 (8 × 94) transformants were screened to identify mutations improving regioselectivity for salidroside or icariside D2. This screening scale represented a dramatic reduction compared to the theoretical requirement of screening 2162 (23 × 94) transformants ‐ the number needed if we had performed SSM across all 23 residues in the binding pocket.

### 2.3 Enhancement of Enzymatic Activity by Access Tunnel Engineering

Enzyme access tunnels that connect the external environment to the active site, play a pivotal role in determining catalytic efficiency by facilitating the efficient transport of substrates, solvents and products to and from the catalytic center.^[^ ^35^ ^]^ The geometry, physicochemical properties and dynamic of tunnels significantly influence the catalytic properties by determining the exchange rates of substrate and products. Tan et al. modified the tunnel entrance of the glycosyltransferase UGT78D2 from *Arabidopsis thaliana* by introducing hydrophobic interactions, resulted in a 10‐fold increase in the binding affinity for the substrate etoposide aglycone.^[^ ^36^ ^]^ Wu et al. enhanced the catalytic efficiency for 2‐CM by 3‐fold through modifications of the twisted access tunnel.^[^ ^37^ ^]^ Seo et al. increased the turnover number of the monooxygenase by 2.8‐fold compared to the wild‐type enzyme through access tunnel engineering;^[^ ^38^ ^]^ Kong et al. achieved a remarkable 42‐fold increase in the enzyme activity of an epoxide hydrolase by substituting bulky residues in the access tunnels.^[^ ^39^ ^]^

Thus, to further improve the catalytic activity of mutants N61I/I62T/L70W (M1) and F111E/M112D (M1‐1) for salidroside and icariside D2 synthesis, respectively, tunnels were identified in both mutants using Caver 3.0 software. Through 100 ns MD simulations, we identified the tunnels for substrate entry (Tunnel 1) and product release (Tunnel 2). During substrate entry into the binding pocket and product release, the substrate and product interact with residues within the tunnel. To elucidate important interactions between reactant molecules and amino acids lining these tunnels, free energy landscape (FEL) analysis was performed using MD trajectories (Figure S6, Supporting Information). Similarly, the RMSD and Rg values of tunnels were used as reaction coordinates to describe the FELs. During substrate entry and product release, the reactants transition from metastable states or transition‐state conformations to the thermodynamically lowest‐energy wells, as mapped by free energy landscape analysis. This suggests that the substrate and product must overcome energy barriers imposed by amino acid interactions within the tunnel to facilitate efficient substrate and product release processes. Interaction analysis, including hydrophobic interactions, hydrogen bond, and π‐π interactions, of the unstable and lowest‐energy conformations identified critical residues in Tunnel 1 (T59, L60, N61, I62, L70, A80, M83, and F84) and Tunnel 2 (L70, K74, P81, E148, E152, P321) that may mediate substrate/product interactions (Figure S7, Supporting Information). Among these residues, L60, N61, I62, and L70 had already been mutated to improve the regioselectivity. Based on these findings, sites T59, K74, A80, P81, M83, E148, E152, and P321 were chosen for further investigation.

Specifically, in an attempt to enhance the catalytic performance of UGT_BL_1, single‐site saturation mutagenesis was conducted on those tunnel residues using mutants M1 (for salidroside production) and M1‐1 (for icariside D2 production) as templates_._ Initial efforts focused on mutating the substrate entry tunnel 1. However, these attempts failed to identify any positive mutants that simultaneously maintained high regioselectivity and improved activity (Figure S8, Supporting Information). This decrease in regioselectivity may result from engineering the substrate entry tunnel, potentially changing the manner in which the substrate accesses to the binding pocket. In contrast, using M1 as the template for mutations in tunnel 2 resulted in six mutants with superior catalytic activity: M1/K74E, M1/E148A, M1/E148M, M1/E152G, M1/E152M, and M1/P321A. From these mutations, M1/K74E, which has g a 1.14‐fold compared to M1, was used to generate double mutants including either the E148A, E148M, E152G, E152M or P321A mutations as well (Figure 4c). The double mutants M1/K74E/E148M and M1/K74E/E152M demonstrated a ≈1.2‐fold activity improvement compared to the M1/K74E mutant, and the triple mutant M1/K74E/E148M/E152M (labelled M2, Figure S9a, Supporting Information) exhibited a remarkable 10.2‐fold increase over the WT. Finally, mutant M2 demonstrated remarkable catalytic activity, and achieved near‐perfect regioselectivity (99.2%), serving as a potential enzyme template for the *de novo* synthesis of salidroside (Figure 3e,f; Tables S3 and S4 and Figures S18, S19, S25–S27, Supporting Information).

Similarly, using M1‐1 as template to introduce mutations in tunnel 2 resulted in seven mutants that maintained high regioselectivity while improving catalytic activity (i.e., M1‐1/P81V, M1‐1/P81N, M1‐1/E148A, M1‐1/E148V, M1‐1/E152G, M1‐1/P321A, and M1‐1/P321V; Figure 4d). Among these, mutant M1‐1/P81N, which has a 2.6‐fold improvement in activity relative to M1‐1, was selected as template for subsequent combinatorial experiments. The combination of P81N with E148A, E152G, and P321A showed no distinct change in activity compared to M1‐1/P81N. However, the introduction of P321V into M1‐1/P81N (resulting in a variant labelled M2‐1, Figure S9b, Supporting Information) resulted in a 5.1‐fold increase in activity relative to M1‐1/P81N, which amounts to 2.7‐fold improvement relative to wild‐type UGT_BL_1 (Figure 4e,f; Tables S3 and S4 and Figures S18, S20, and S28–S30, Supporting Information). Thus, our data demonstrate that results the engineering of enzyme access tunnel is an effective strategy for enhancing catalytic performance.

### 2.4 Biochemical Characterizations of UGT_BL_1 Variants

The enzymatic activity of WT and its variants M2 and M2‐1 were investigated at the temperature range from 20 °C to 50 °C and the pH range from 5.0 to 10.0, respectively. The three enzymes exhibit an optimal temperature of 30 °C (Figure S10a, Supporting Information). Variants M2 is thermally more stable than wild‐type UGT_BL_1, maintaining above 80% activity after a 2‐h incubation at 40 °C compared to ≈70% for the later (Figure S10b, Supporting Information). Variant M2‐1, in contrast, has reduced thermal stability, maintaining <40% activity under these experimental conditions. In terms of optimal pH for activity and pH stability, there is no significant difference between the three enzymes. They all exhibit maximum activity in phosphate buffer at pH 8.0 (Figure S10c, Supporting Information) and maintain at least 20% of their maximal activity from pH 5.0 to pH 10.0 (Figure S10d, Supporting Information).

Kinetic analysis of WT and its variants M1, M1‐1, M2 and M2‐1 were investigated in detail using tyrosol and UDPG as substrates (**Table** **1**; Figure S11, Supporting Information). Mutants M1 and M2 are both efficient in the synthesis of salidroside. M1, obtained by reshaping cavity B in the substrate binding pocket, showed improved substrate affinity, evidenced by a decreased *K*_m_ value (2.60 mm) compared to that of the wild‐type enzyme (4.32 mM). The mutant is also considerably more reactive (with *k*_cat_ values of 1.13 and 0.22 s^−1^, respectively), resulting in an 8.8‐fold increased catalytic efficiency (*k*_cat_/*K*_m_) for M1. Adding mutations in tunnel 2 to M1 to generate mutant M2 leads to a further improvement of catalytic rate (*k*_cat_ of 1.45 s^−1^), which constitutes a 14.8‐fold higher catalytic efficiency when compared to wild‐type UGT_BL_1. Hence, modification of the product release pathway (tunnel 2) leads to significant improvements in the overall performance of the enzyme. Total turnover numbers (TTNs) were determined to assess the performance of WT and the two mutants, with M1 (549.41) and M2 (850.00) having significantly higher than the WT (79.72) (Table 1). This also indicates that these variants simultaneously improved both regioselectivity and catalytic performance.

**Table 1:** Catalytic activity of wild‐type UGT_BL_1 and four of its mutants toward substrate tyrosol.

*Enzyme*	*Activity* [U mg^−1^]	*K*_m_ [mm]	*k*_cat_ [s^−1^]	*k*_cat_/*K*_m_ ^a^ ^)^ [s^−1^ ·mm^‐1^ ]	*TTN*
**WT**	0.04	4.32 ± 0.42	0.22	0.05	79.72
**M1**	0.27	2.60 ± 0.24	1.13	0.44	549.41
**M2**	0.41	1.96 ± 0.15	1.45	0.74	850.00
**M1‐1**	0.01	3.59 ± 0.65	0.12	0.03	16.32
**M2‐1**	0.11	2.92 ± 0.13	0.53	0.18	200.46

^a)^ Kinetic parameters were determined in 200 µL reaction mixtures including 100 µg of purified enzymes, 50 mM phosphate buffer (pH 8.0), 5 mm UDPG with varying concentrations of tyrosol (0–7 mM) at 30 °C for 1 h.

^b)^ Total turnover numbers (TTN): [product formation] / [enzyme]. TTNs were determined with 100 µg of purified enzymes, 2 mm tyrosol, 5 mm UDPG, 2% DMSO and 2 mm MgCl_2_ in a total volume of 1 mL at 30 °C for 16 h.

Mutants M1‐1 and M2‐1 have been shown to be efficient in for the regioselective synthesis of icariside D2 (see above). For mutant M1‐1, similar to mutant M1, the reshaping of substrate binding pocket (i.e., cavity A) has a beneficial impact on substrate binding when compared to wild‐type UGT_BL_1 (*K*_m_ of 3.59 mm vs 4.32 mm). However, in contrast to M1, the improved substrate binding in M1‐1 comes at the cost of significantly reduced activity (*k*_cat_ of 0.12 s^−1^ vs 0.22 s^−1^), which results in an overall loss of the catalytic efficiency by 40%. But, as the data in Table 1 indicate, modifying M1‐1 by introducing mutations in tunnel 2 leads to significant improvements in catalytic rate (k_cat_ of 0.53 s^−1^), resulting in a 3.6‐fold improvement of the catalytic efficiency when compared to the wild‐type enzyme. This improvement is also reflected in the TTN of M2‐1, which is ≈2.5‐fold higher than that of the wild‐type. The significant improvements observed for both M2 and M2‐1 demonstrate that improving product release (by engineering tunnel 2; Figure S8, Supporting Information) has a profound effect on their catalytic performance.

To evaluate the sugar donor promiscuity of WT and its mutants, four common UDP‐sugar donors, including UDP‐α‐D‐Gal, UDP‐α‐D‐Xyl, UDP‐α‐D‐GlcA, and UDP‐α‐D‐GalA, were assayed using tyrosol as the sugar acceptor (Figure S12, Supporting Information). The wild‐type enzyme demonstrated activity with UDP‐α‐D‐Gal and UDP‐α‐D‐Xyl. Mutants M2 and M2‐1 retained the sugar donor promiscuity, maintaining catalytic activity with UDP‐Gal, and UDP‐Xyl (Figures S21–S24, Supporting Information). No products were observed when WT, M2 and M2‐1 were used as biocatalysts with either UDP‐α‐D‐GlcA or UDP‐α‐D‐GalA as sugar donors. These findings suggest that the regioselective modification of acceptor did not affect the sugar donor preference.

### 2.5 Molecular Mechanism Contributing to the High Regioselectivity and Catalytic Activity of Mutant M2

**Figure 5:**
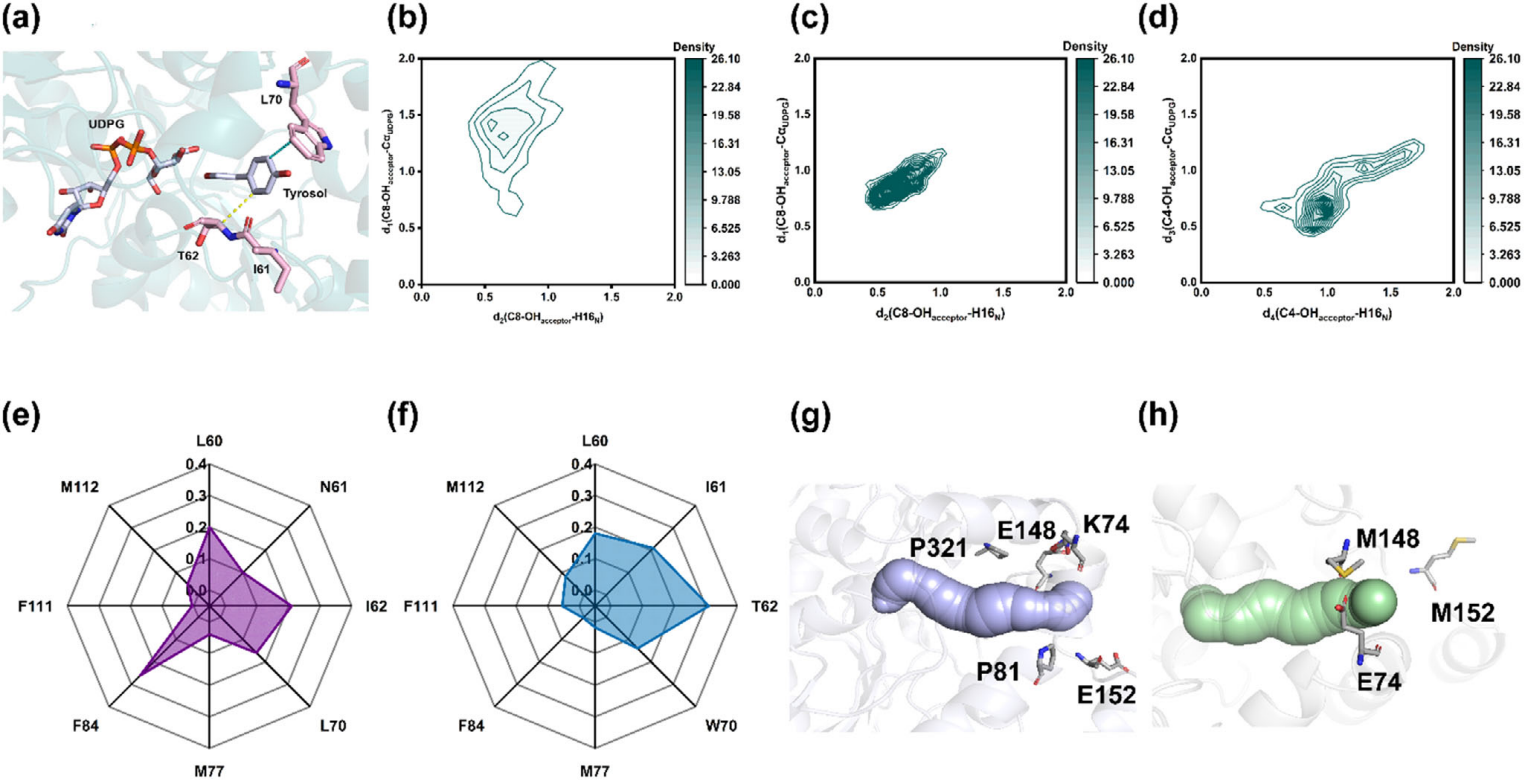
Analysis of the factors that contribute to improved regioselectivity and activity of variant M2. a) Representative snapshot of ligands bound in the substrate binding cavity. π–π interactions are depicted by blue dotted lines. Hydrophobic interactions are shown as yellow dotted lines. b–d) Analysis of PRSs. The distance distribution between the C4‐OH and C8‐OH of tyrosol and the Cα atom of UDPG and the N atom of H16. The conformation satisfying both d_1_ and d_2_ <3.5 Å is used for calculating the population of PRSs. The attack frequency is defined as a spatial proximity metric quantifying the spatial closeness. b) C8/B‐WT; c) C8/B‐M2; d) C4/B‐WT. e,f) Protein‐ligand IFPs for wild‐type e) UGT_BL_1 and f) variant M2. The IFP radar plots depict the statistical protein‐ligand interaction frequencies obtained from 100 ns MD simulations. g,h) Profiles of tunnel 2 in (g) wild‐type UGT_BL_1 and h) mutant M2; hotspots surrounding this product release tunnel are shown as sticks.

To elucidate the structural basis for the high regioselectivity and activity of the mutant toward the C8‐OH moiety of tyrosol, 100 ns MD simulations were performed for two complexes: tyrosol in the form of C8‐OH and UDPG in cavity B of the WT for salidroside synthesis (designated as C8/B‐WT), and tyrosol in the form of C8‐OH and UDPG in cavity B of mutant M2 (designated as C8/B‐M2). The root‐mean‐square deviations (RMSDs) of the C*α* atoms of two complexes stabilized after 75 ns (Figures S13 and S5d, Supporting Information). From a representative snapshot, in the complex C8/B‐M2, the side chain of T62 introduces a new hydrophobic interaction with tyrosol, and the substitution of L70 with tryptophan establishes a new π‐π stacking interaction with the benzene ring of tyrosol (**Figure** **5**a). These interactions likely facilitate the appropriate positioning of tyrosol for the production of the C8‐OH product, salidroside. Together with I61, T62, and W70 shape the hydrophobic microenvironment of the substrate binding pocket, which is consistent with reported studies that suggested that introduction of hydrophobic amino acids surrounding the substrate contribute to the change of regioselectivity.^[^ ^2^, ^40^ ^]^ Thus, the synergistic effect of the aforementioned mutations in positions 61,62, and 70 of WT leads to greatly favored C8‐OH orientation for tyrosol in cavity B of M2, promoting excellent regioselectivity of C8‐OH.

In addition, MD simulations were performed to compare the distance C8‐OH of tyrosol to the anomeric carbon atom (Cα) of UDPG and H16 in the complexes C8/B‐M2 and C8/B‐WT. For the C8/B‐M2 complex the distance between C8‐OH and Cα is smaller and the attack frequency between C8‐OH and Cα higher than in the corresponding C8/B‐WT complex. Similarly, the distance between C8‐OH and nitrogen atom of H16 in C8/B‐M2 is smaller and the associated attack frequency higher than in the C8/B‐WT complex. The combined data are in agreement with the observed strongly improved regioselectivity of the mutant in favor of salidroside production. While in wild‐type UGT_BL_1 the B/C4‐OH orientations appear to be favored, which leads to the production of icariside D2 (see above), the reshaping of cavity B led to a drastic change in the preferred binding orientation of tyrosol in that location in favor of salidroside production (Figure 5b,c). Our findings are in good agreement with studies engineering of other glycosyltransferases that reducing the distance between reactive sites improves regioselectivity significantly. For example, Cui et al. engineered the glycosyltransferase *Sg*UGT94‐289‐3 from *Siraitia grosvenorii* and found that in the V148F mutant, the position of the 2‐hydroxyl group of substrates was closer to both the Cα of UDPG and catalytic residue. This proximity facilitated the precise positioning of the 2‐OH group within the active pocket, thereby enhancing the regioselectivity for 2‐OH.^[^ ^40^ ^]^ He et al. revealed that the spatial proximity between the C8 position of apigenin/luteolin and the Cα of UDPG promotes regioselective glycosylation at the C8 site through structural analysis of glycosyltransferase *Tc*CGT1 from *Trollius chinensis*.^[^ ^41^ ^]^

In order to gain insight into the high catalytic efficiency of mutant M2, the C8/B‐M2 complex was compared with the complex of wild‐type UGT_BL_1 where tyrosol is oriented in the C4‐OH orientation to favor icariside D2 synthesis (C4/B‐WT complex). The root‐mean‐square deviation (RMSD) of the C*α* atoms of C4/B‐WT and C8/B‐M2 complexes stabilized after 75 ns (Figures S13 and S5c, Supporting Information). Structural overlap of WT and M2 models does not reveal noticeable conformational changes in most residues surrounding tyrosol, except for the three sites that were mutated (i.e., 61, 62, and 70; (Figure S14a, Supporting Information). Pocket calculations showed that the surface area of the active site of M2 was 3019.95 Å^2^, compared to 2851.75 Å^2^ for the WT. Furthermore, the volumes of the substrate binding pocket of two enzymes are 2944.10 Å^3^ and 2762.05 Å^3^, respectively (Figure S15a,b, Supporting Information). These findings lead to the conclusion that the larger pocket volume and surface area in M2 may facilitate greater substrates to accessibility, thereby enhancing catalytic efficiency. This observation is consistent with the findings of Guo et al. who demonstrated that the reshaping of the hydrophobic pocket allows for increased substrate accommodation, ultimately improving the catalytic efficiency of the glycosyltransferase UGT76G1.^[^ ^42^ ^]^ Protein‐ligand interaction fingerprint (IFP) analysis provides atomic‐level insights into the interactions between active substrates and residues in the binding pocket (Figure 5e,f).^[^ ^43^, ^44^ ^]^ For instance, an IFP analysis of carboxylic acid reductase identified interactions between residues in binding pocket and the substrate that are crucial for substrate recognition and the catalytic efficiency.^[^ ^44^ ^]^ Here, a radar chart illustrating contact frequencies between the enzyme and ligand was generated for both WT and variant M2 to highlight differences in the probability of relevant residues to interact with tyrosol. In particular the residues in position 61 and 62 exhibit increased frequency of interactions in mutant, in excellent with our experimental data, which are thought to enhance the recognition between the enzyme and substrate, thus contributing to the improved catalytic efficiency. These findings align with those of Han et al., who indicated that substitution at glycosylation sites increase the number of hydrogen bonds between glycosyltransferase CGTase and substrate. Such enhanced interactions improve the binding capacity thereby improving catalytic efficiency.^[^ ^45^ ^]^

The pre‐reaction state (PRS) is described as the conformation of the enzyme‐substrate complex immediately preceding the critical transition state along the reaction coordinate, providing direct insights into the fit between enzyme and substrate.^[^ ^46^, ^47^ ^]^ For inverting glycosyltransferases, the prereaction state is defined by two thresholds: the distance between the O_acceptor_ and H16_N_ < 3.5 Å and distance between O_acceptor_ and Cα of UDPG < 3.5 Å.^[^ ^48^ ^]^ The probability of prereaction‐state formation was calculated as the proportion of MD simulation conformations that simultaneously satisfied both distance criteria (Figure S16, Supporting Information). To evaluate the potential of pre‐reaction states, we focused again on the complexes C4/B‐WT and C8/B‐M2. MD simulations showed that in C8/B‐M2 the distance d_1_ (C8‐OH_acceptor_‐ Cα_UDPG_) is shorter than the corresponding distance d_3_ (C4‐OH_acceptor_‐ Cα_UDPG_) in C4/B‐WT. Similarly, distance d_2_ (C8‐OH_acceptor_‐H16_N_) in C8/B‐M2 is shorter than d_4_ (C4‐OH_acceptor_‐H16_N_) in C4/B‐WT (Figure 5c,d). These results indicate that in cavity B the C8‐OH orientation in mutant M2 is more favorable for the reaction compared to the C4‐OH in the WT. Moreover, the proportion of the near‐attack conformation in M2 is higher than in WT. These observations suggest that mutant M2 maintains superior catalytic efficiency due to its ability to readily forms PRS and supporting a higher frequency for the nucleophilic attack than the WT. This study aligns with Li et al., who employed PRS calculations to assess the frequency of catalytic conformations of glycosyltransferases UGT74AC1 from *Siraitia grosvenorii* and demonstrated that appropriate distances promote catalysis, thereby increasing glycosylation activity.^[^ ^49^ ^]^

Modifications to enzyme access tunnel are known to be an effective strategy to improve catalytic efficiency of enzymes.^[^ ^37^ ^]^ As shown in Figure 5g,h, the entrance of tunnel 2 was enlarged due to the substitutions at positions 74 (K74E), 148 (E148M), and 152 (E152M) in variant M2. The more open entrance likely to facilitate a higher rate of product release. A tunnel analysis, including throughput, length, and curvature, was performed to shed further light on the improved catalytic performance. The introduction of the mutations decreases the average curvature in the mutant from 1.27 Å to 1.23 Å, indicating that the tunnel changed to a less twisted (i.e., more straightened) conformation. Furthermore, the throughput value increased from 0.59 to 0.63, and the tunnel length was shortened from 23.3 Å to 22.4 Å, both consistent with a faster rate of product transportation, thereby improving catalytic efficiency (Table S2, Supporting Information).

In summary, the structural comparisons between of mutant M2 and WT provide insights into the factors that contribute to both regioselectivity and catalytic efficiency. The introduction of specific mutations favors the positioning of the C8‐OH of tyrosol, enhancing interactions with tyrosol and promoting the formation of favorable pre‐reaction states. Additionally, modifying the product tunnel accelerates the rate of product release. The findings illustrate that rational design glycosyltransferases can effectively control regioselectivity and increase catalytic performance.

### 2.6 Molecular Mechanism Contributing to The Improved Regioselectivity and Catalytic Activity of Mutant M2‐1

**Figure 6:**
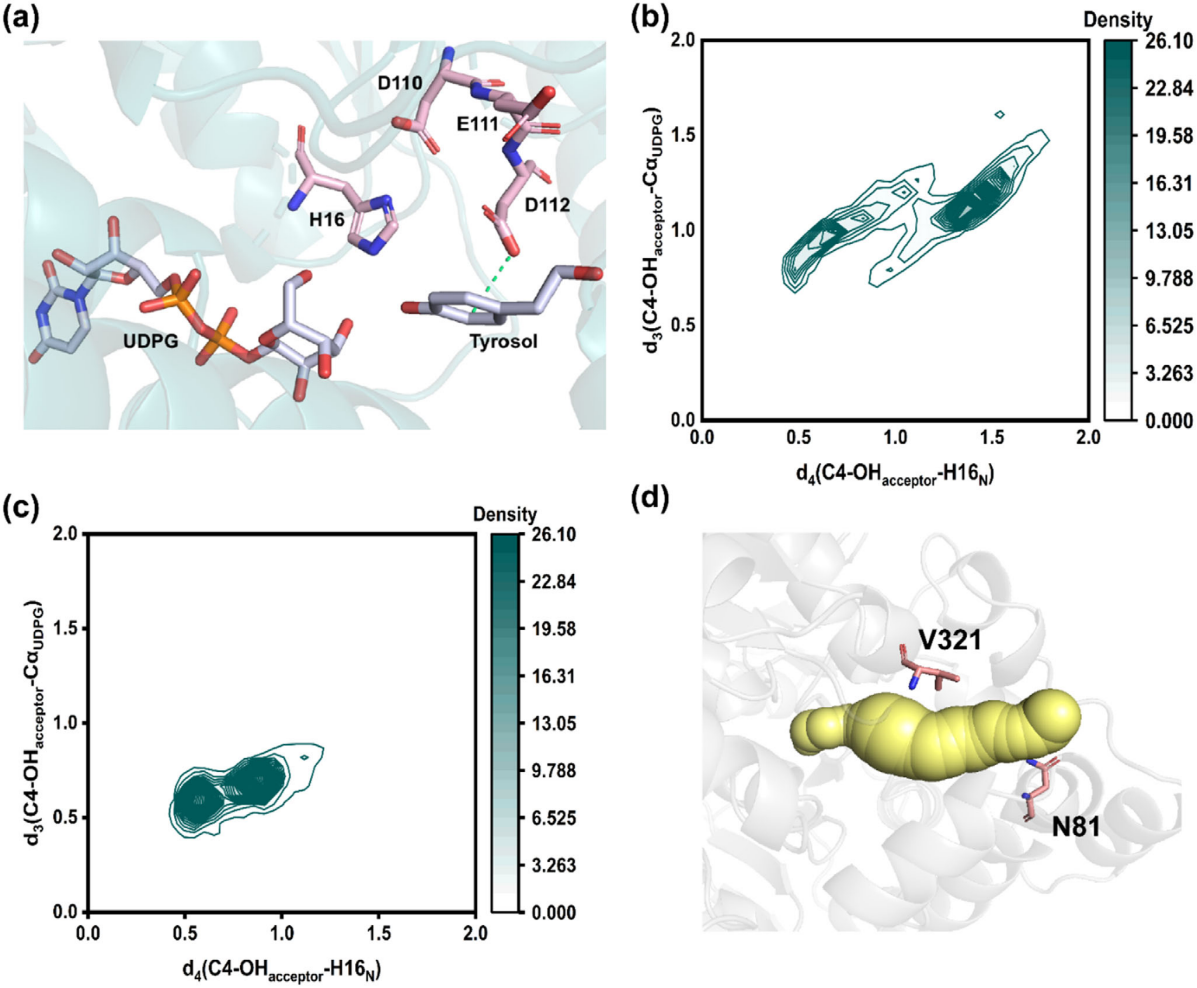
Analyses of the factors that contribute to improved regioselectivity of variant M2‐1. a) Representative snapshot of ligands bound in the substrate binding cavity. Pi‐Aion interactions are depicted by green dotted lines. b,c) Analysis of PRSs. The distance distribution between the C4‐OH of tyrosol and the Cα atom of UDPG and the N atom of H16. b) C4/A‐WT; c) C4/A‐M2‐1. d) The profile of tunnel of M2‐1; residues that were mutated are depicted as sticks.

To elucidate the structural basis for improved the C4‐OH glycosylation (icariside D2 synthesis) of this variant a similar approach as discussed in the previous section was applied. We performed comparative MD simulations of two complexes: tyrosol in the form of C4‐OH and UDPG in cavity A in WT (C4/A‐WT) and mutant M2‐1(C4/A‐M2‐1). Structural overlap of WT and M2‐1 reveal noticeable conformational changes in the binding pocket with the exception of the two mutation sites in cavity A described above (*i*.*e*. F111E and M112D; Figure S14b, Supporting Information). The RMSD of the C*α* atoms of the C4/A‐WT and C4/A‐M2‐1 complexes stabilized after 75 ns (Figures S13 and S5a, Supporting Information). The M112D mutation, located near the catalytic residues H16 and D110, is likely to facilitate a pi‐Anion interaction between the aspartate in position 112 and the benzene ring of tyrosol, which anchors C4‐OH in a position suitable for attack on the glucose group of UDPG. The F111 residue creates significant steric hindrance due to the presence of its phenyl ring, affecting the interaction between tyrosol and UDPG. Mutating it to glutamate eliminates unfavorable steric contacts, together with D112, stabilizes the appropriate conformation for icariside D2 synthesis (**Figure** **6**a).

To validate this interpretation, comparative MD simulations were conducted to assess the relative positions of the C4‐OH of tyrosol, the anomeric carbon atom (Cα) of UDPG and H16 in the complexes C4/A‐WT and C4/A‐M2‐1. In the latter, which produces icariside D2, the distance between C4‐OH and Cα is smaller and the attack frequency between them is higher than in C4/A‐WT. Additionally, the distance between C4‐OH and the nitrogen atom of H16 in C4/A‐M2‐1 is also smaller and the attack frequency between them greater than the corresponding values in C4/A‐WT (Figure 6b,c). Collectively, these observations demonstrate that the C4‐OH of tyrosol in the M2‐1 mutant is indeed better positioned for effective glycosylation than in the C4/A‐WT complex. While cavity A in the wild‐type enzyme prefers glycosylation at the C8‐OH group (i.e., salidroside formation), the M2‐1 mutant has shifted its regioselectivity almost completely toward the C4‐OH group (i.e., icariside D2 formation).

In contrast to mutant M2, where the mutations in the substrate binding cavity led to both a preferred regioselectivity and improved catalytic efficiency, in mutant M2‐1 the success in regioselectivity is accompanied by a significant loss in catalytic efficiency. The volume and surface area of the substrate binding pockets in wild‐type (2762.05 Å^3^ and 2851.75 Å^2^) and mutant M2‐1 (1395.2 Å^3^ and 1635.91 Å^2^) are largely different, with the latter exhibiting a considerable contraction (Figure S15c, Supporting Information). While this more limited space may contribute to regiospecific binding, it may also limit structural flexibility needed for the glycosylation reaction, leading to reduced catalytic efficiency. Note that in mutant M2 (see above), the size of the substrate binding pocket is slightly larger than in the wild‐type enzyme.

Tunnel analysis indicated that replacing rigid P81 and P321 with asparagine and valine, respectively, led to a large entrance that is similar to that in the M2 mutant (Figure 6d). The results of tunnel calculation also reveal that the mean curvature of the mutant decreased from 1.27 to 1.20 Å, suggesting a partial straightening of the twisted structure, as observed in mutant M2‐1. Furthermore, the throughput value increased from 0.59 to 0.65, while the tunnel length contracted from 23.3 to 20.6 Å (Table S2, Supporting Information). These changes reflect an enhanced rate of product transportation, which corresponds well with the observed improvement in catalytic efficiency (Table 1). Hence, our strategy to simultaneously modify residues in the substrate binding pocket and the product release tunnel enable us to engineer mutants UGT_BL_1 that are highly selective in regards of substrate binding, enabling the efficient production of either salidroside or icariside D2 with nearly perfect specificity.

### 2.7 Whole‐Cell Reactions for Producing Salidroside or Icariside D2

The conventional in vitro enzymatic synthesis of salidroside or icariside D2 faces significant limitations in industrial‐scale applications, primarily due to its dependence on expensive UDPG as substrate. In light of this, our study explores an alternative approach using whole‐cell biocatalysis with engineered glycosyltransferase mutants M2 and M2‐1, which exhibit enhanced catalytic performance. The time course of the salidroside or icariside D2 synthesis was monitored under optimal conditions (Figure S17, Supporting Information). Using a cell density of OD_600_ at 5 and tyrosol concentration of 2 g/L, the engineered mutants achieved remarkable productions. Salidroside reached a final concentration of 1.99 g/L, with a 3.2‐fold increase over the wild‐type (0.62 g/L). Icariside D2 accumulated to 1.22 g/L, representing a 2.0‐fold improvement compared to the WT (0.59 g/L). These results highlight the superior catalytic efficiency of the M2 and M2‐1 mutants, demonstrating their potential as robust biocatalysts for large‐scale production. Notably, this whole‐cell system eliminates the need for costly UDPG supplementation by leveraging endogenous UDP‐glucose production within the recombinant cells, offering a promising alternative to traditional in vitro enzymatic methods.

## 3 Conclusion

The spacious substrate binding pocket in UGT_BL_1 enables the binding of tyrosol in two distinct conformations, thus facilitating the transfer of glucose to two different acceptor groups within this substrate. Principal component analysis of the free energy landscape of the protein‐ligand complexes guided our structural engineering efforts, leading to both the reshaping of substrate binding pocket and the modification of the product release tunnel. These alterations collectively addressed the inherent challenges associated with regioselectivity and catalytic efficiency without the need for expansive screening experiments. Specifically, we successfully generated two mutants, M2 and M2‐1, which demonstrate exceptional regioselectivity of 99.2% and 99.1% toward C8‐OH and C4‐OH, respectively. The two variants also exhibit remarkable improvement in catalytic efficiency, achieving 14.8‐fold and 3.6‐fold enhancements in catalytic efficiency compared to wild‐type UGT_BL_1. The molecular basis for these improvements was investigated with comprehensive MD simulations, including protein‐ligand interactions, PRS and substrate tunnel analysis. This study demonstrated that only a small number of mutations are needed to engineer glycosyltransferases with exceptional regioselectivity to produce valuable compounds.

## Conflict of Interest

The authors declare no conflict of interest.

## Supporting information



Supporting Information

## Data Availability

The data that support the findings of this study are available from the corresponding author upon reasonable request.
