# Supplementary material for: Tailoring Regioselectivity‐Controlled UDP‐Glycosyltransferase for Bidirectional Glycosylation of Tyrosol via Free Energy‐Driven Pocket Reshaping and Tunnel Engineering
Source: Adv Sci (Weinh). 2025 Aug 4;12(40):e09814. doi: 10.1002/advs.202509814 (PMC12561194; doi:10.1002/advs.202509814)
Supplement: Supplementary file 1 — Supporting Information [file ADVS-12-e09814-s001.docx]

**Tailoring Regioselectivity-controlled UDP-Glycosyltransferase for Bidirectional Glycosylation of Tyrosol *via* Free Energy-driven Pocket Reshaping and Tunnel Engineering**

Ziyu Zhang^1^, Jingyi Chen^1^, Zuozhi Liang^1^, Wenming Shao^1^, Zhen Gao*^1^, Bin Wu*^1^, Bingfang He^2^, Gerhard Schenk^3^

^1^ College of Biotechnology and Pharmaceutical Engineering, Nanjing Tech University, 30 Puzhunan Road, Nanjing 211816, China, 202462118232@njtech.edu.cn (Z.Z), 202261218219@njtech.edu.cn (J.C), 202461118040@njtech.edu.cn (Z.L), 202362181216@ njtech.edu.cn (W.S).

^2^ School of Pharmaceutical Sciences, Nanjing Tech University, 30 Puzhunan Road, Nanjing 211816, China.

^3^ School of Chemistry and Molecular Biosciences, The University of Queensland, Brisbane, QLD, 4072, Australia.

Correspondence: gaozhen_njtech@163.com, wubin1977@njtech.edu.cn

**Supporting information**

**1 Materials and methods**

**1.1 Chemicals, Materials and Strains**

Salidroside, icariside D2 and tyrosol were purchased from Dingguo Biotech (Tianjin, China). Uridine diphosphate glucose (UDPG) and all of the other reagents used in this study were obtained from Sigma-Aldrich Chemical Co., Ltd (StLouis, MO, USA), Aladdin Biochemical Technology Co., Ltd (Shanghai, China) and Sinopharm Chemical Reagent Co., Ltd (Shanghai, China). Restriction enzymes and phanta max super-fidelity DNA polymerase were purchased from Vazyme Biotech Co., Ltd (Nanjing, China). *E. coli* DH5α cells and PMD19-T vector for DNA manipulations and plasmid maintenance, and *E. coli* BL21 (DE3) cells and the pET-28a (+) for expression of recombinant proteins were preserved in our laboratory.

**1.2 Molecular Docking and Molecular Dynamics Simulations of UGT_BL_1 with Tyrosol**

The three-dimensional structure model of UGT_BL_1 was constructed using AlphaFold 2.^[1]^ The structures of UDPG and tyrosol were obtained from PubChem (<https://pubchem.ncbi.nlm.nih.gov>). Molecular docking of UGT_BL_1 with its ligands was performed using Discovery Studio 2019 (Biovia, San Diego, CA, USA). Before docking, the structure of the protein was pretreated using the “Prepare protein” module of Discovery Studio 2019 by eliminating all bound water molecules and adding hydrogen atoms. The ligands were also optimized by adding hydrogen atoms at pH 8.0 and energy minimized by the “Prepare ligand” module of Discovery Studio 2019. UDPG was imported into the reaction center with the reference conformation of 7VLB and then tyrosol was docked into UGT_BL_1 with the CDOCKER program of Discovery Studio 2019. Visualization and figure construction of the protein and complex structures were performed using Pymol (Version 2.4.1, Schrodinger LLC). The best scoring pose was selected for structural feature analysis and molecular dynamic simulations.

MD simulations were carried out using GROMACS 5.1.4. The protein was described by the Amber99sb forcefield and ligands were assigned to the Amber GAFF forcefield.^[2]^ By adding TIP3P water molecules and 2% DMSO, and the protein-ligand complex after docking was solvated in a cubic box with periodic XYZ boundary conditions of 10 × 10 × 10 nm^3^. The solvent environment was modeled explicitly with certain numbers of counter ions (Na^+^ and Cl^-^) for simulating the real reaction environment. Prior to MD simulations, energy minimization was conducted using the conjugate gradient method. Subsequently, energy minimizations were performed with the steepest descent algorithm. The simulation parameters were set as follows: a step size of 0.001 nm, 50,000 steps, and minimization convergence criteria defined by a maximum force of less than 500 kJ mol^-1^ nm^-1^. Thereafter, the system was heated to 300 K and at a pressure of 1 atm for 100 ps, and the timestep was 2 fs to carry out the temperature and pressure simulations. Finally, 100 ns MD simulations were performed for each protein-ligand system. Hydrogen bonds were constrained using the SHAEK algorithm. The Particle Mesh Ewald (PME) method was applied to calculate long-range electrostatic interactions.^[3]^ The convergences were evaluated by root-mean-square deviation (RMSD) analysis. Pymol was used to visual protein-ligand interaction profiles and generate illustrative figures.

**1.3 Construction of Mutant Library and Screening**

The codon of the selected site was replaced by the NNK degeneracy codon using the QuickChange method. pET28a-UGT_BL_1 was employed as the template and the primers used are listed in Table S1. The PCR products were digested with the restriction enzyme *Dpn* I and transformed into *E. coli* DH5α cells to obtain the desired mutants. Subsequently, the intended mutations were confirmed by DNA sequencing and the corresponding plasmids were used to transform into *E. coli* BL21 for protein expression. The colonies were cultures in 96 deep-well-plates (2.2 ml) containing 450 μL LB liquid medium with 50 μg/ml kanamycin sulfate at 37 ℃. The cells were induced with 0.1 mM IPTG when the OD_600_ reached 0.6. After induction at 20 ℃ for 16 h, the cells were harvested by centrifugation at 8000 rpm for 10 min, and then washed by phosphate buffer (50 mM, pH 8.0). Subsequently, the harvested cells were suspended in 250 μL of phosphate buffer and frozen at -80 ℃ for 12 h. The frozen 96-deep-well plate were carefully thawed in a water bath until the mixture melted completely. After that, the cells were disrupted with lysozyme. The resulting mixture was centrifuged at 5000 rpm for 10 min. Initial screening of enzymatic activity used crude extracts of the mutants. SDS-PAGE confirmed comparable expression of the target enzyme across mutants. The protein concentration of crude enzyme was determined by Bradford assay. Subsequently, mutants that exhibited ​enhanced activity relative to the wild-type​​ in the crude extract screening were ​subjected to protein purification. Enzymatic activity was then re-assessed using the purified proteins to confirm the observed increases and to ensure the reliability of the results. The glycosylation of tyrosol was performed in a total of 200 μL phosphate buffer containing 2 mM tyrosol, 5 mM UDPG and 50 μL of the supernatant at 30 ℃ for 1 hour. The reaction was quenched by the addition of 800 μL methanol and analyzed by high performance liquid chromatography (HPLC).

**1.4 Heterogonous Expression and Purification of UGTs**

Recombinant *E. coli* BL21 expressing UGT_BL_1 or mutants was cultured in LB medium supplement with 50 μg/ml kanamycin. Recombinant protein expression was triggered by adding IPTG as described above. The overexpressed recombinant enzyme was purified in a single step by affinity chromatography using a HisTrap FF column (GE Health, Fairfield, USA) according to the manufacture’s manual. The apparent molecular weight and homogeneity of the purified enzymes were estimated on a 12% SDS-PAGE gel. The concentration of the proteins was measured using the Bradford assay. The activity and regioselectivity were assayed using wild-type UGT_BL_1 as control.

**1.5 Activity and Regioselectivity Assay**

The activities of UGT_BL_1 and its mutants were assayed in 50 mM potassium phosphate buffer (pH 8.0) containing 2 mM tyrosol, 5 mM UDPG and an appropriate amount of purified enzyme. The reactions were performed at 30 ℃ for 1 h and then quenched with methanol. Subsequently, the reactants were centrifuged and filtered through a 0.22 μm membrane and analyzed by HPLC. Then, concentrations were calculated from the peak area of the glycosylation product. One unit of enzyme activity (U) was defined as the enzyme amount able to synthesize 1 μmol of glucoside products per minute.

**1.6 Biochemical Characterizations of UGT_BL_1 Mutants**

The optimal pH for UGT_BL_1 (WT) and its mutants was determined at 30 ℃ in a pH range from 5-10 using citric acid/sodium citrate (pH 5-6), sodium phosphate buffer (pH 6-8) and Tris-HCl (pH 8-10). The optimal temperature for the enzymes was evaluated from 20 ℃ to 50 ℃ under standard conditions. To estimate their pH stability and thermal stability, the enzymes were preincubated at various pH values at 4 ℃ for 2 h and different temperatures at pH 8.0 for 2 h, following which the remaining activity was determined under standard conditions. The apparent kinetic constants the enzymes were determined under optimal temperature and pH conditions with varying concentrations of tyrosol (0-7 mM). The data were analyzed using GraphPad Prism 9.0 (GraphPad Software, La Jolla, CA, USA). The kinetic parameters, *i*.*e*., the Michaelis constant (*K*_m_), the turnover rate (*k*_cat_) and catalytic efficiency (*k*_cat_/*K*_m_) were obtained by fitting the experimental data to the Michaelis-Menten equation.

To determine the specificity of wild-type, as well as mutants M2 and M2-1 towards various glycosyl acceptors, the reaction mixture containing 50 mM potassium phosphate buffer (pH 8.0), 2 mM glycosyl acceptors, 5mM UDPG, and 50 μg/ml purified enzymes, was agitated at 30 ℃ for 6 h, and then quenched by methanol. The resulting mixtures were subjected to HPLC, high-resolution mass spectrometry (HR-MS) and NMR analyses.

**1.7 Molecular Dynamic Simulations of UGT_BL_1 and Its Mutants**

The homology models of UGT _BL_1 mutants were constructed using AlphaFold 2. Molecular docking and MD were performed using the same settings as the described above. The trajectory file was written every 5000 steps with an integration time -step of 2 fs, and it was then analyzed using the trajectory protocol. The analysis of the prereaction states were performed with the last 1000 frames of the trajectories. The parameters of the substrate-binding pocket and binding free energy between the enzyme and substrate were calculated for 50 snapshots extracted from the last 1000 frames of the trajectories. The access tunnels of UGT_BL_1 and its mutants were analyzed by CaverDock.^[33]^ Taking the active center as the starting point of the CAVER calculation, the minimum probe radius, shell depth, shell radius, clustering threshold, maximum distance, and desired radius were set to 0.9, 4, 3, 3.5, 3 and 5 Å, respectively.

**1.8 Preparative-Scale Reactions for Salidroside and Icariside D2 synthesis**

Preparative-scale reactions were performed at a 100 ml-scale by whole-cell bioconversion. The reaction mixture contained the whole cells (wet cell loading) of the engineered mutants or UGT_BL_1, tyrosol (2 g/L), glucose (40 g/L), DMSO (5%, v/v) and potassium phosphate buffer (50 mM, pH 8.0). The reaction was performed at 30 ℃ and 200 rpm for 24 h. Sample (100 μl) were intermittently collected and subjected to HPLC analysis for conversion determination. After that, the mixture was centrifuged at 4 ℃ at 12000 rpm for 10 min, and then the supernatant was extracted with an equivalent volume of butanol. The crude product was further purified by reverse-phase semi-preparative HPLC on a column (SinoChrom ODS-BP 10μm, 4.6 mm*250mm). The mobile phase consisted of water and methanol in a ratio of 89:11 with a flow rate of 1 ml/min. The purified glycosylation products were subjected to LC/MS and nuclear magnetic resonance (NMR) analysis for structure identified.

**1.9 Analytic Methods**

**1.9.1 HPLC analysis**

The conversion analysis was performed *via* reversed-phase HPLC using Piomex P680 (Sunnyvale, CA, USA) equipped with a C18 column (Kromasil, 4.6*250mm, 5μm, Bohus, Sweden). The column temperature and flow rate were 30 ℃ and 1.0 ml/min, respectively. The mobile phase consisted of water and methanol in a ratio of 80:20, and detection was conducted at 278 nm. For quantification of the glycosylation products, samples were diluted as required and the standard calibration curves were generated with a series of known concentration of the standard.

**1.9.2 LC-MS analysis**

Liquid chromatography-mass spectrometry (LC-MS) analysis was performed in positive ion mode on an Agilent 6520 Accurate-MassQ-TOF LC/MS platform (Palo Alto, CA, USA).

**1.9.3 NMR analysis**

NMR spectra were recorded for ^1^H NMR at 500 MHz, ^13^C NMR at 125 MHz and HMBC spectrum at 500 MHz and 125 MHz respectively on a Bruker Avance III HD spectrometer.

**Salidroside**

**
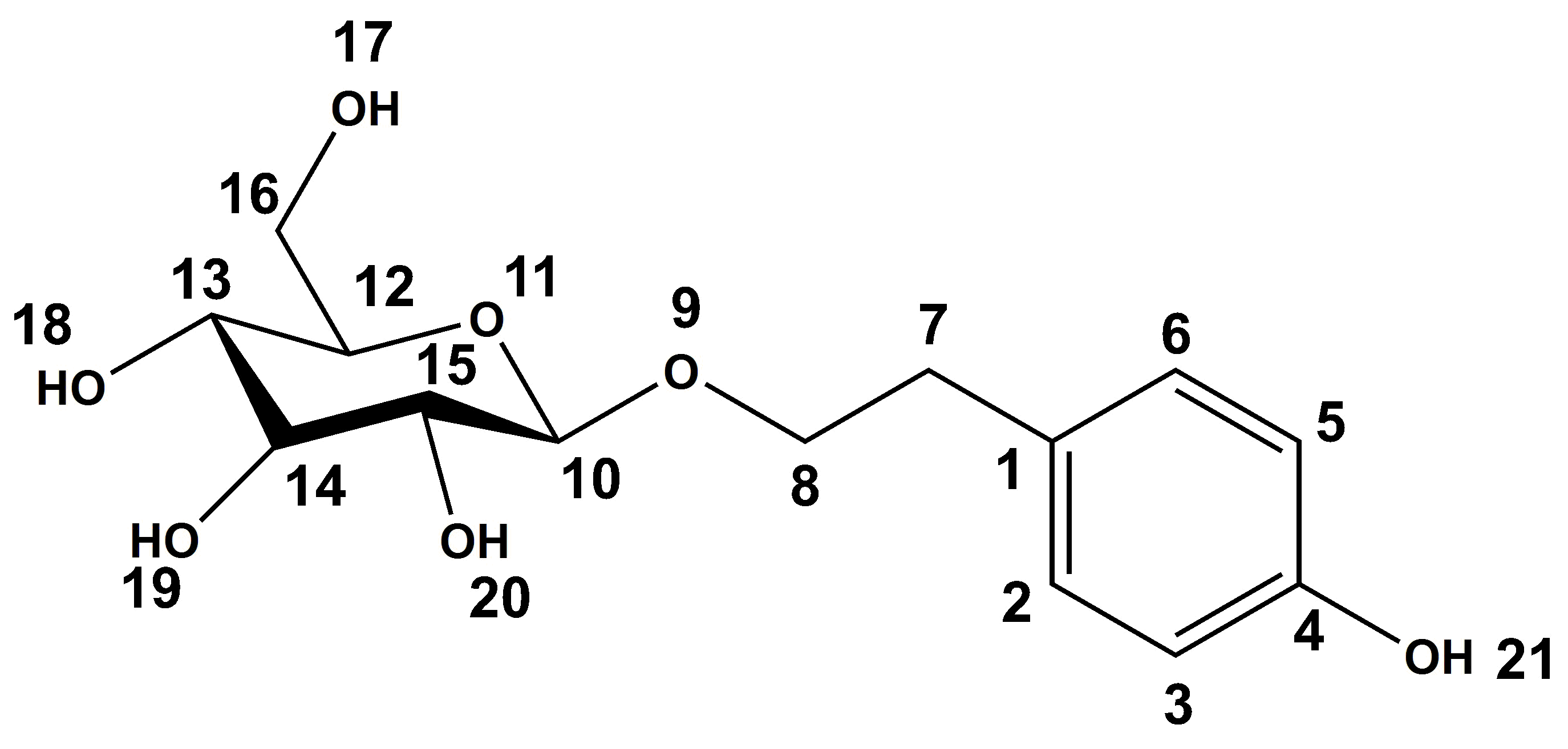
**

**^1^H NMR** (500 MHz, DMSO-d6) δ 9.14 (s, 1H), 7.04 (d, J = 8.2 Hz, 2H), 6.66 (d, J = 8.4 Hz, 2H), 4.96 (d, J = 5.0 Hz, 1H), 4.91 (d, J = 4.8 Hz, 1H), 4.87 (d, J = 5.1 Hz, 1H), 4.47 (t, J = 5.9 Hz, 1H), 4.16 (d, J = 7.8 Hz, 1H), 3.87 (td, J = 9.0, 6.7 Hz, 1H), 3.66 (ddd, J = 11.7, 6.0, 2.1 Hz, 1H), 3.56 (td, J = 9.1, 6.4 Hz, 1H), 3.43 (dt, J = 11.7, 5.8 Hz, 1H), 3.19 - 2.99 (m, 3H), 2.95 (ddd, J = 8.9, 7.8, 4.9 Hz, 1H), 2.80 - 2.66 (m, 2H).

**^13^C NMR** (126 MHz, DMSO) δ 155.62, 129.77, 128.60, 115.03, 102.85, 76.90, 76.80, 73.46, 70.10, 69.92, 61.10, 34.84.

**HMBC** ^13^C NMR (126 MHz, DMSO) δ 156.14, 156.14, 76.65, 101.06, 76.65, 156.14, 39.56, 62.85, 62.85, 130.31, 62.85, 130.31.

^1^H NMR (500 MHz, DMSO) δ 7.12, 6.93, 5.26, 5.26, 5.06, 4.80, 4.60, 4.60, 3.69, 3.58, 2.68, 2.68.

**Icariside D2**

**
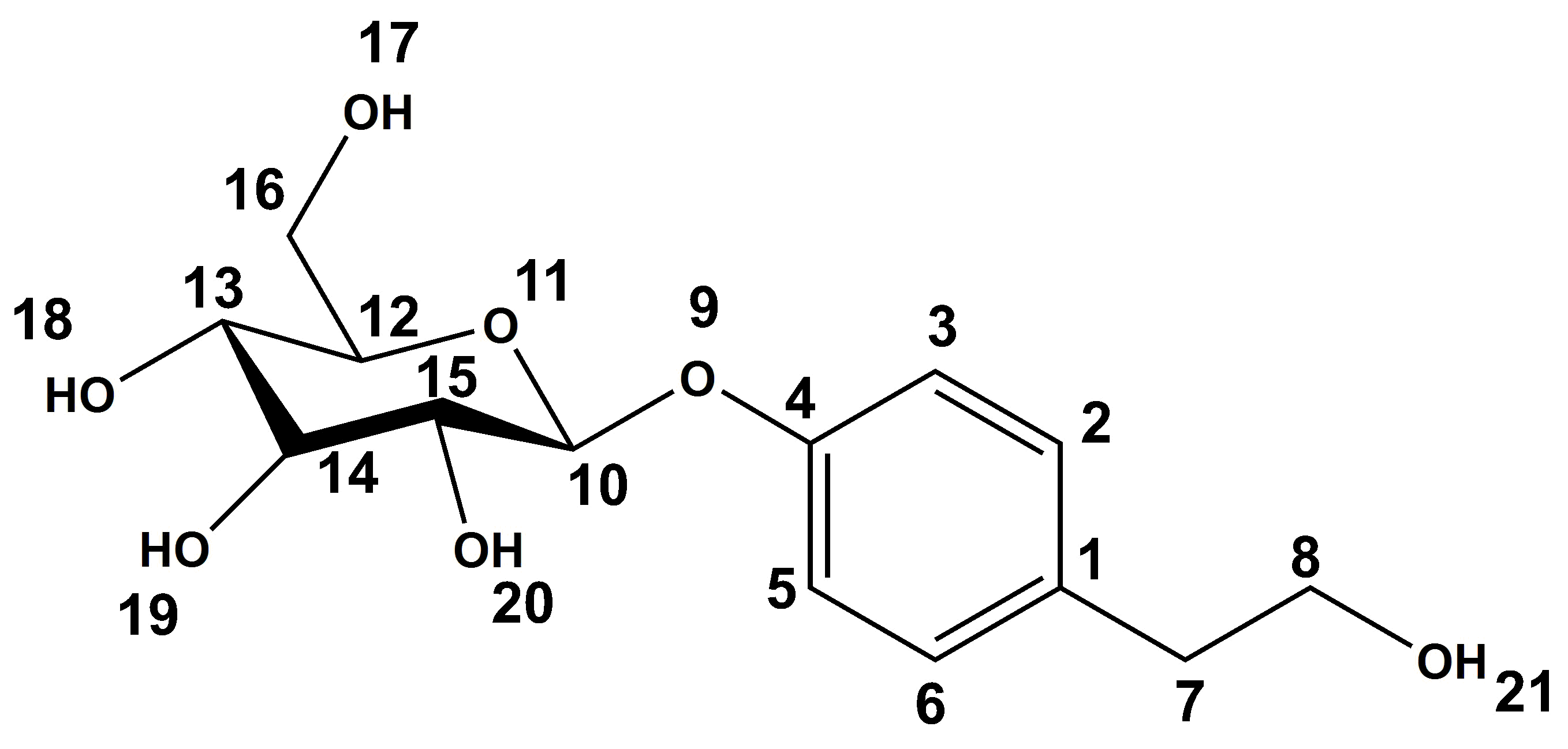
**

**^1^H NMR** (500 MHz, DMSO-d_6_) δ 7.11 (d, J = 8.6 Hz, 2H), 6.93 (d, J = 8.6 Hz, 2H), 5.26 (d, J = 4.9 Hz, 1H), 5.06 (d, J = 4.7 Hz, 1H), 5.00 (d, J = 5.2 Hz, 1H), 4.79 (d, J = 7.5 Hz, 1H), 4.59 (t, J = 5.2 Hz, 1H), 4.57 - 4.51 (m, 1H), 3.68 (ddd, J = 11.8, 5.3, 2.1 Hz, 1H), 3.55 (td, J = 7.2, 5.2 Hz, 2H), 3.45 (dt, J = 11.9, 6.0 Hz, 1H), 3.31 - 3.11 (m, 4H), 2.65 (t, J = 7.1 Hz, 2H).

**^13^C NMR** (126 MHz, DMSO) δ 155.76, 132.75, 129.65, 116.06, 100.59, 77.00, 76.64, 73.25, 69.74, 62.40, 60.72, 38.21.

**HMBC** ^13^C NMR (126 MHz, DMSO) δ 62.44, 130.17 ^1^H NMR (500 MHz, DMSO) δ 2.60, 2.60.

**2 Interpretation of Informatics Results:**

Our structural informatics and MD data can be directly interpreted as follows:

**2.1 Free Energy Landscape (FEL) Analysis**

Key structural parameters (e.g., backbone RMSD, radius of gyration (Rg)) were extracted from MD trajectories to construct a 2D free energy distribution map, enabling evaluation of the major conformational states of the system. The global minimum corresponds to the most stable bound conformation (energy lowest point), while local minima represent potential metastable states that may associate with catalytic intermediate states.

**2.2 Principal Component Analysis (PCA)**

By analyzing the global trajectory, high-dimensional conformational data was reduced to principal components (PC1/PC2). PCA was applied to capture the dominant conformational fluctuation modes, thereby elucidating the coordinated motions of key residues and regions.

**2.3 Protein-ligand interaction fingerprint (IFP) analysis**

IFPs represent interactions between protein residues and ligand atoms as binary bits (0/1) or numerical values, capturing specific interaction types (e.g., hydrogen bonds, hydrophobic contacts).

**Supporting Tables**

**Table S1.** Primers used in this work.

| Amino acid sites | Primers (5’→3’) |
| --- | --- |
| L60 | F: CTACCGCTCAACT**NNK**AATATCGATCCGCAGCAAATTC |
|  | R: T**MNN**AGTTGAGCGGTAGTTGAGCGGCTCTGCC |
| N61 | F: A**NNK**ATCGATCCGCAGCAAATTCGGGAGCTGA |
|  | R: GCTGCGGATCGAT**MNN**TAAAGTTGAGCGGTAGTTGAGCG |
| I62 | F: AAAT**NNK**GATCCGCAGCAAATTCGGGAGCTGA |
|  | R: GCTGCGGATC**MNN**ATTTAAAGTTGAGCGGTAGTTGAGC |
| L70 | F: GCAAATTCGGGAG**NNK**ATGAAAAATAAAAAGGATATGACACA |
|  | R: T**MNN**CTCCCGAATTTGCTGCGGATCGATATTT |
| M77 | F: GGAT**NNK**ACACAGGCTCCGATGATGTTTATGA |
|  | R: GAGCCTGTGT**MNN**ATCCTTTTTATTTTTCATCAGCTCC |
| F84 | F: GGCTCCGATGATG**NNK**ATGAAAGAAATGGAGGAGGTTCTTC |
|  | R: T**MNN**CATCATCGGAGCCTGTGTCATATCCTTT |
| F111 | F: TGAC**NNK**ATGGCCATGGCGGGAAAAATGCTGG |
|  | R: CCATGGCCAT**MNN**GTCAAAAAGGATGAGGTCAGGC |
| M112 | F: GACTTT**NNK**GCCATGGCGGGAAAAATGCTGGC |
|  | R: GCCATGGC**MNN**AAAGTCAAAAAGGATGAGGTCAGG |
| N61I/I62 | F: TAATC**NNK**GATCCGCAGCAAATTCGGGAGCTG |
|  | R: CTGCGGATC**MNN**GATTAAAGTTGAGCGGTAGTTGAGC |
| N61I/I62T/L70 | F: GCAAATTCGGGAG**NNK**ATGAAAAATAAAAAGGATATGACACA |
|  | R: T**MNN**CTCCCGAATTTGCTGCGGATCGATAATT |
| N61I/I62T/L70W/M77 | F: AAGGAT**NNK**ACACAGGCTCCGATGATGTTTAT |
|  | R: GCCTGTGT**MNN**ATCCTTTTTATTTTTCATCCATCCC |
| F111E/M112 | F: TTGACGAG**NNK**GCCATGGCGGGAAAAATGCTG |
|  | R: CATGGC**MNN**CTCGTCAAAAAGGATGAGGTCAG |
| M1/K74 | F: T**NNK**AAGGATATGACACAGGCTCCGATGATGT |
|  | R: GTGTCATATCCTT**MNN**ATTTTTCATCCACAGCTCCCG |
| M1/P81 | F: CACAGGCT**NNK**ATGATGTTTATGAAAGAAATGGAGGA |
|  | R: CATCAT**MNN**AGCCTGTGTCATATCCTTTTTATTTTT |
| M1/E148 | F: A**NNK**TTTAAGATCGAGCTCACGCCTGAGCAAG |
|  | R: GCTCGATCTTAAA**MNN**TTCAGACATTGATTTGAATGAAAAATG |
| M1/E152 | F: AAGATC**NNK**CTCACGCCTGAGCAAGAAGCCGC |
|  | R: GGCGTGAG**MNN**GATCTTAAACTCTTCAGACATTGATTTGA |
| M1/P321 | F: GCAAATG**NNK**GAACAGGAAATCACTGCCCGCC |
|  | R: CCTGTTC**MNN**CATTTGCGGGACGGCAACAAGC |
| M1/T59 | F: CAACTACCGCTCA**NNK**TTAATTACCGATCCGCAGCAA |
|  | R: A**MNN**TGAGCGGTAGTTGAGCGGCTCTGCCCCA |
| M1/A80 | F: GACACAG**NNK**CCGATGATGTTTATGAAAGAAATGG |
|  | R: TCATCGG**MNN**CTGTGTCATATCCTTTTTATTTTTCATC |
| M1/M83 | F: CAGGCTCCGATG**NNK**TTTATGAAAGAAATGGAGGAGGTTC |
|  | R: AA**MNN**CATCGGAGCCTGTGTCATATCCTTTTT |
| M1/K74E/E148A | F: A**GCG**TTTAAGATCGAGCTCACGCCTGAGCAAG |
|  | R: GCTCGATCTTAAA**CGC**TTCAGACATTGATTTGAATGAAAAATG |
| M1/K74E/E148M | F: A**ATG**TTTAAGATCGAGCTCACGCCTGAGCAAG |
|  | R: GCTCGATCTTAAA**CAT**TTCAGACATTGATTTGAATGAAAAATG |
| M1/K74E/E152G | F: TTAAGATC**GGC**CTCACGCCTGAGCAAGAAGCC |
|  | R: CGTGAG**GCC**GATCTTAAACTCTTCAGACATTGATTTGA |
| M1/K74E/E152M | F: TAAGATC**ATG**CTCACGCCTGAGCAAGAAGCCG |
|  | R: GCGTGAG**CAT**GATCTTAAACTCTTCAGACATTGATTTGA |
| M1/K74E/P321A | F: CAAATG**GCG**GAACAGGAAATCACTGCCCGCCG |
|  | R: TCCTGTTC**CGC**CATTTGCGGGACGGCAACAAG |
| M1/K74E/E148M/E152M | F: TAAGATC**ATG**CTCACGCCTGAGCAAGAAGCCG |
|  | R: GCGTGAG**CAT**GATCTTAAACATTTCAGACATTGATTTG |
| M1-1/K74 | F: T**NNK**AAGGATATGACACAGGCTCCGATGATGT |
|  | R: GTGTCATATCCTT**MNN**ATTTTTCATCAGCTCCCGAATTT |
| M1-1/P81 | F: CACAGGCT**NNK**ATGATGTTTATGAAAGAAATGGAGGA |
|  | R: CATCAT**MNN**AGCCTGTGTCATATCCTTTTTATTTTT |
| M1-1/E148 | F: A**NNK**TTTAAGATCGAGCTCACGCCTGAGCAAG |
|  | R: GCTCGATCTTAAA**MNN**TTCAGACATTGATTTGAATGAAAAAT |
| M1-1/E152 | F: TAAGATC**NNK**CTCACGCCTGAGCAAGAAGCCG |
|  | R: GCGTGAG**MNN**GATCTTAAACTCTTCAGACATTGATTTGA |
| M1-1/P321 | F: CCCGCAAATG**NNK**GAACAGGAAATCACTGCCCG |
|  | R: GTTC**MNN**CATTTGCGGGACGGCAACAAGCGGT |
| M1-1/T59 | F: CAACTACCGCTCA**NNK**TTAAATATCGATCCGCAGCAAA |
|  | R: A**MNN**TGAGCGGTAGTTGAGCGGCTCTGCCCCA |
| M1-1/A80 | F: GACACAG**NNK**CCGATGATGTTTATGAAAGAAATGG |
|  | R: TCATCGG**MNN**CTGTGTCATATCCTTTTTATTTTTCATC |
| M1-1/M83 | F: GGCTCCGATG**NNK**TTTATGAAAGAAATGGAGGAGGTTC |
|  | R: TAAA**MNN**CATCGGAGCCTGTGTCATATCCTTT |
| M1-1/P81N/E148A | F: A**GCG**TTTAAGATCGAGCTCACGCCTGAGCAAG |
|  | R: GCTCGATCTTAAA**CGC**TTCAGACATTGATTTGAATGAAAAATG |
| M1-1/P81N/E148V | F: A**GTG**TTTAAGATCGAGCTCACGCCTGAGCAAG |
|  | R: GCTCGATCTTAAA**CAC**TTCAGACATTGATTTGAATGAAAAATG |
| M1-1/P81N/E152G | F: TTAAGATC**GGC**CTCACGCCTGAGCAAGAAGCC |
|  | R: CGTGAG**GCC**GATCTTAAACTCTTCAGACATTGATTTGA |
| M1-1/P81N/P321V | F: GCAAATG**GTG**GAACAGGAAATCACTGCCCGCC |
|  | R: CCTGTTC**CAC**CATTTGCGGGACGGCAACAAGC |
| M1-1/P81N/P321A | F: GCAAATG**GCG**GAACAGGAAATCACTGCCCGCC |
|  | R: GCTCGATCTTAAA**CGC**TTCAGACATTGATTTGAATGAAAAATG |

**Table S2.** Tunnel analysis of wild-type UGT_BL_1 (WT) and its mutants M2 and M2-1.

| Name | Throughput | Length(Å) | Curvature(Å) |
| --- | --- | --- | --- |
| WT | 0.59 | 23.3 | 1.27 |
| M2 | 0.63 | 22.4 | 1.23 |
| M2-1 | 0.65 | 20.6 | 1.20 |

**Table S3.** ^1^H NMR Data for compounds 1a and 1b.

| **No.** | **1a** | **1b** |
| --- | --- | --- |
| **1** | - | - |
| **2** | 6.66, d | 7.11, d |
| **3** | 7.04, d | 6.93, d |
| **4** | - | - |
| **5** | 7.04, d | 6.93, d |
| **6** | 6.66, d | 7.11, d |
| **7** | 2.80 - 2.66, m | 2.65, t |
| **8** | 3.87, td  3.56, td | 3.31 - 3.11, m |
| **9** | - | - |
| **10** | 4.16, d | 5.26, d |
| **11** | - | - |
| **12** | 2.95, ddd | 3.45. dt |
| **13** | 3.19 - 2.99, m | 3.55, td |
| **14** | 3.43, dt | 3.55, td |
| **15** | 3.66, ddd | 3.68, ddd |
| **16** | 3.19 - 2.99, m | 3.31 - 3.11, m |
| **17** | 4.47, t | 4.59, t |
| **18** | 4.91, d | 4.57 - 4.51, m |
| **19** | 4.16, d | 5.00, d |
| **20** | 4.87, d | 5.06, d |
| **21** | 9.14, s | 4.79, d |

**Table S4.**^13^C NMR Data for compounds 1a and 1b.

| **No.** | **1a** | **1b** |
| --- | --- | --- |
| **1** | 128.6 | 132.75 |
| **2** | 129.77 | 129.65 |
| **3** | 115.03 | 116.06 |
| **4** | 155.62 | 155.76 |
| **5** | 115.03 | 116.06 |
| **6** | 129.77 | 129.65 |
| **7** | 34.84 | 38.21 |
| **8** | 69.92 | 60.72 |
| **9** | - | - |
| **10** | 102.85 | 100.59 |
| **11** | - | - |
| **12** | 76.9 | 77 |
| **13** | 70.1 | 69.74 |
| **14** | 76.8 | 76.64 |
| **15** | 73.46 | 73.25 |
| **16** | 61.1 | 62.4 |
| **17** | - | - |
| **18** | - | - |
| **19** | - | - |
| **20** | - | - |
| **21** | - | - |

**Supporting Figures**

**
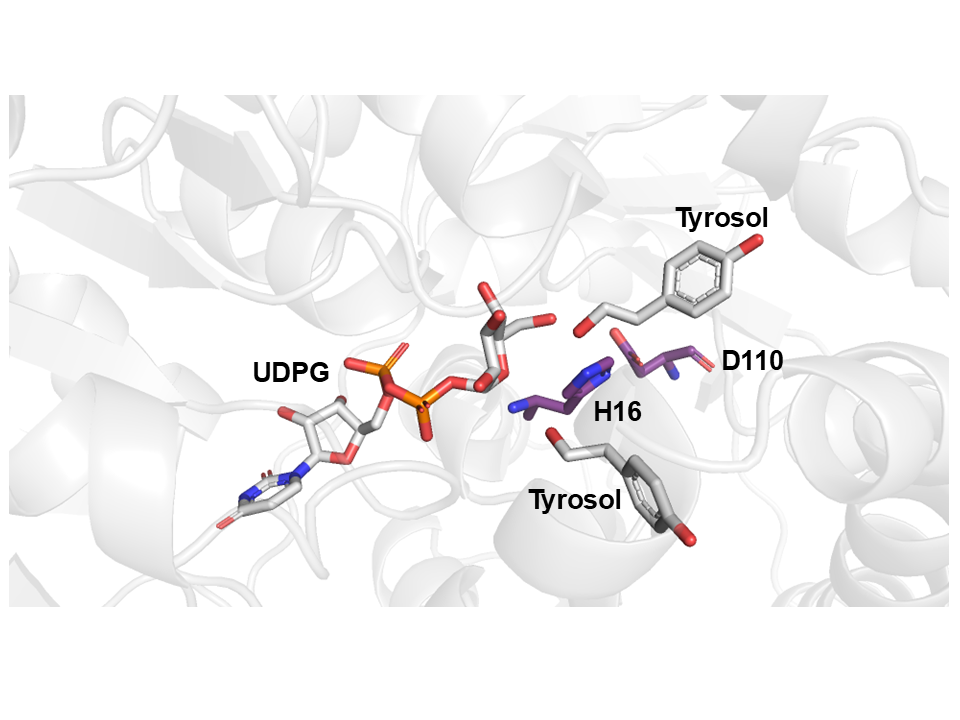
**

**Figure S1.** Catalytic residues of UGT_BL_1. Key residues H16 and D110 are shown as sticks.


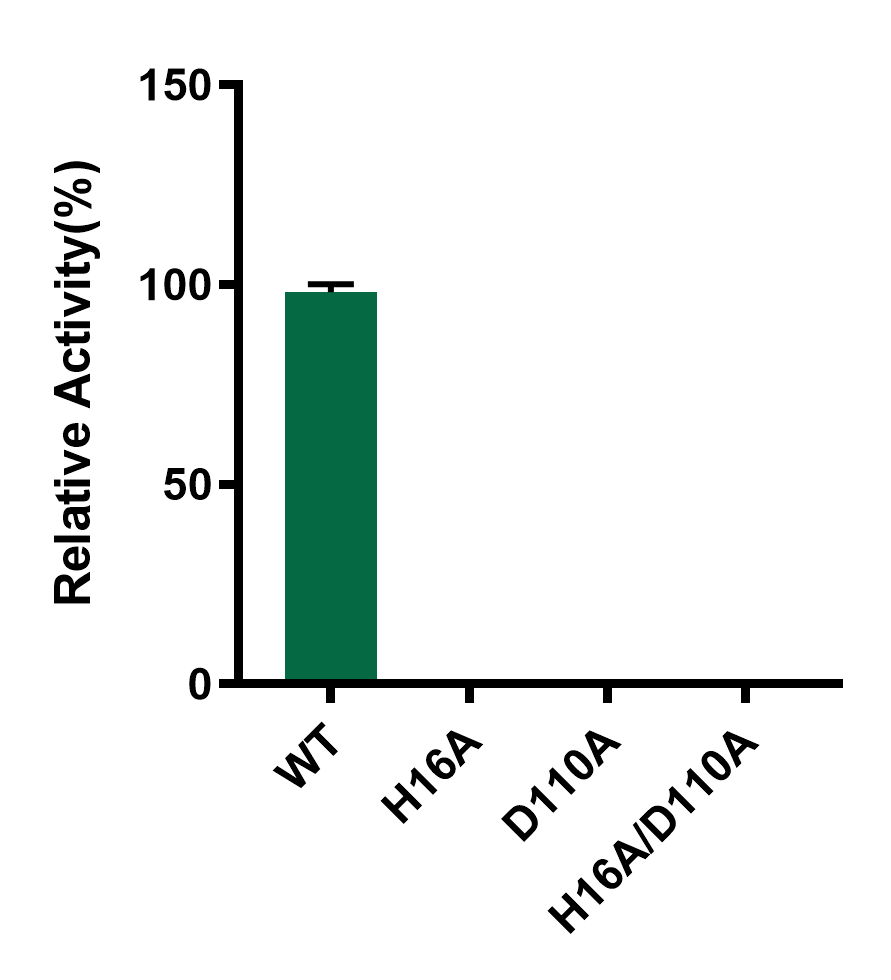


**Figure S2.** Specific activity of UGT_BL_1 wild type (WT) and mutants H16A and D110A in catalytic sites. The data represent mean *±* SD of three independent replicates (n = 3).


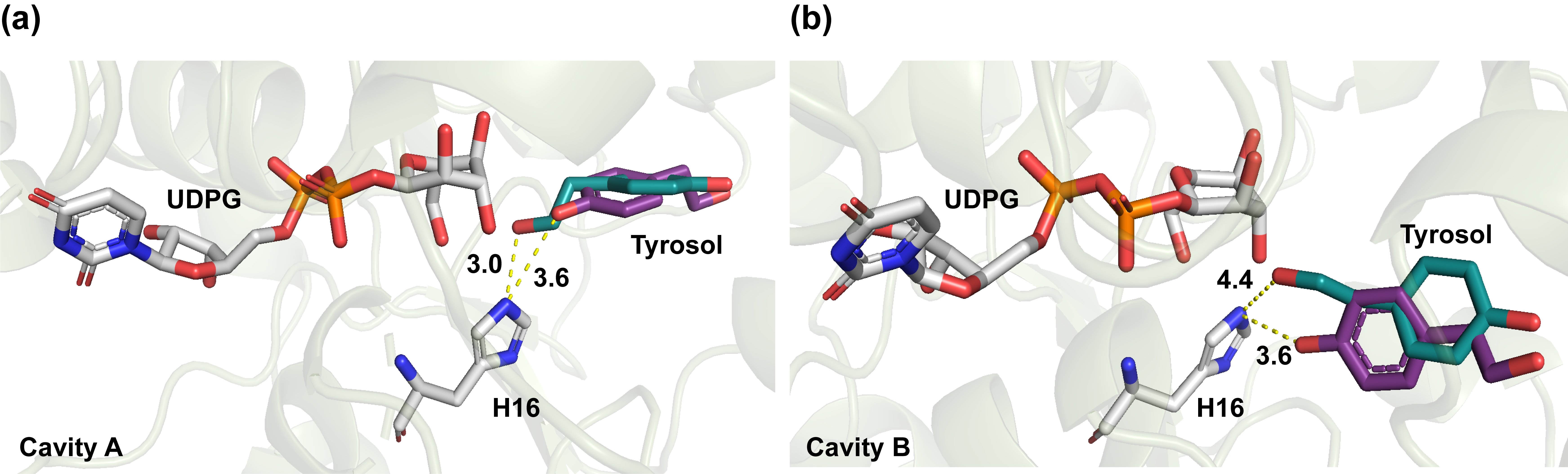


**Figure S3.** The distance between alcoholic and phenolic hydroxyl groups of tyrosol and catalytic residue H16 in cavity A and cavity B.


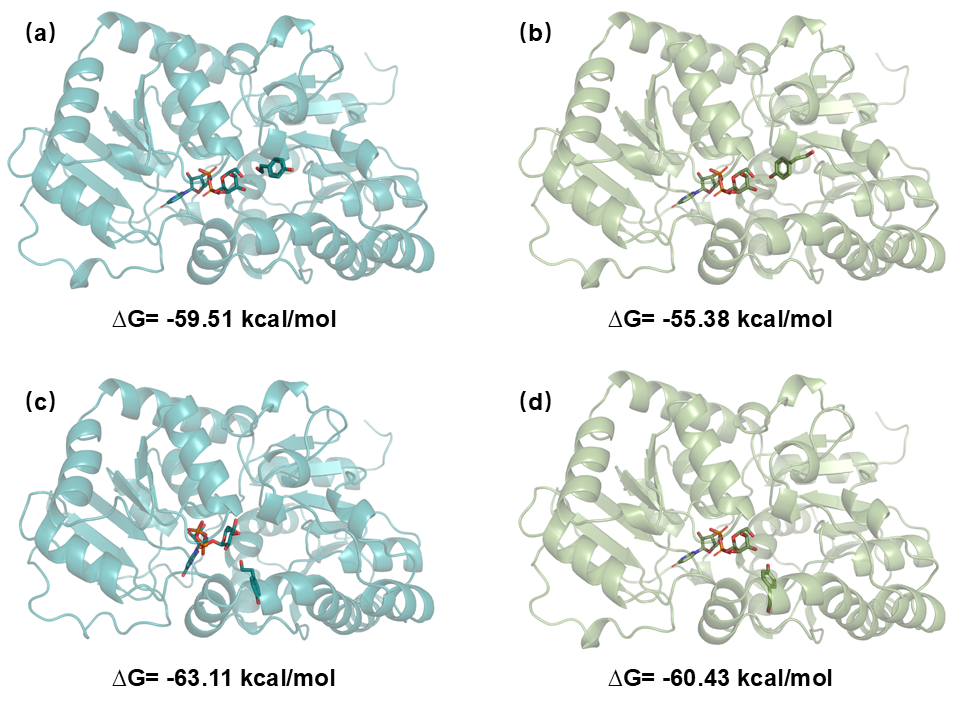


**Figure S4.** The docking poses and binding energy of tyrosol in the pocket of wild-type. (a) The docking binding energy of tyrosol in the form of C8-OH in cavity A. (b) The docking binding energy of tyrosol in the form of C4-OH in cavity A. (c) The docking binding energy of tyrosol in the form of C8-OH in cavity B. (d) The docking binding energy of tyrosol in the form of C4-OH in cavity B.


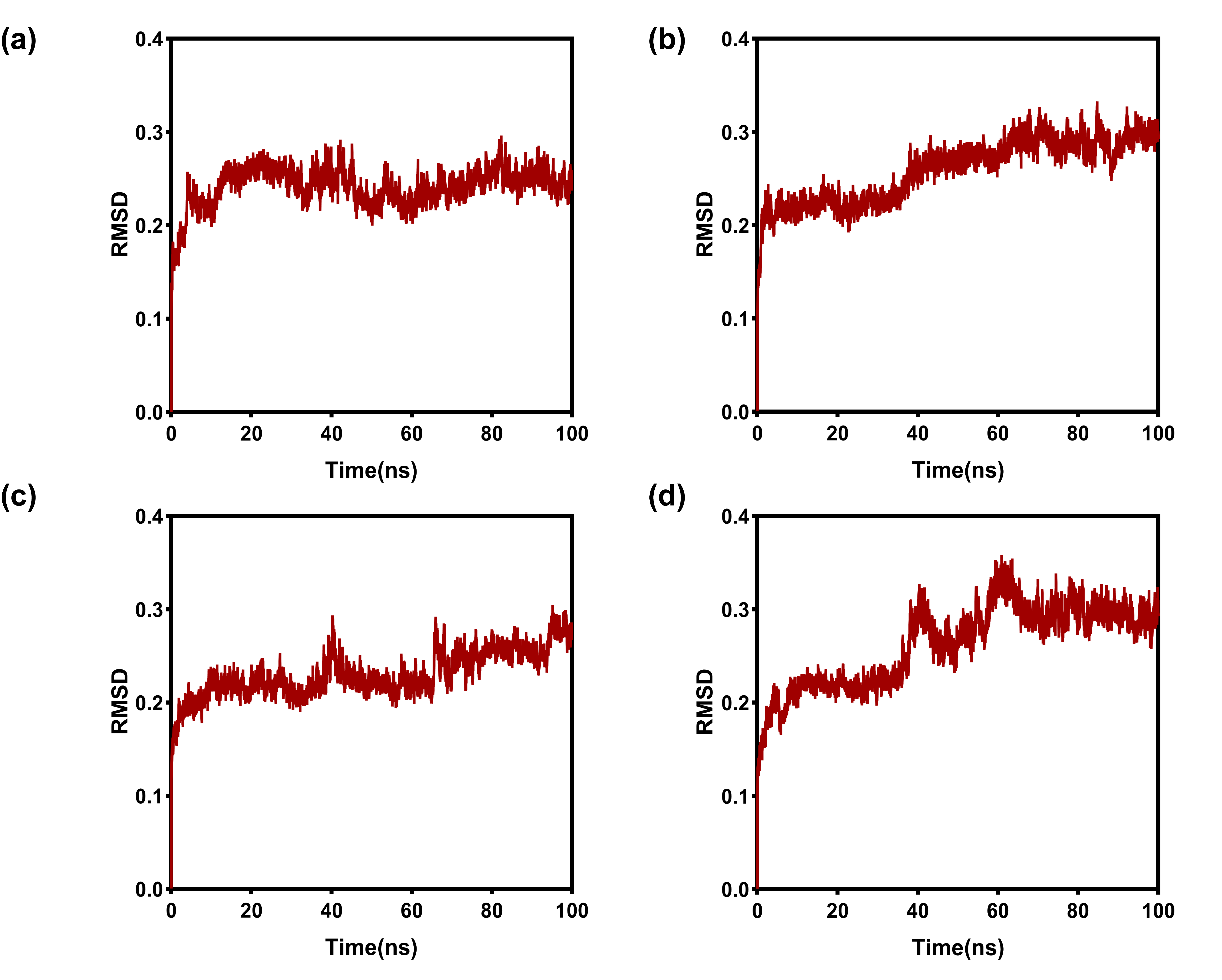


**Figure S5**. The Root-mean-square deviation (RMSD) of four docking results during 100 ns MD simulations. (a) The RMSD of complex with UDPG and tyrosol in the form of C4-OH in cavity A (C4-OH/A). (b) The RMSD of complex with UDPG and tyrosol in the form of C8-OH in cavity A (C8-OH/A). (c) The RMSD of complex with UDPG and tyrosol in the form of C4-OH in cavity B (C4-OH/B). (d) The RMSD of complex with UDPG and tyrosol in the form of C8-OH in cavity B (C8-OH/B).


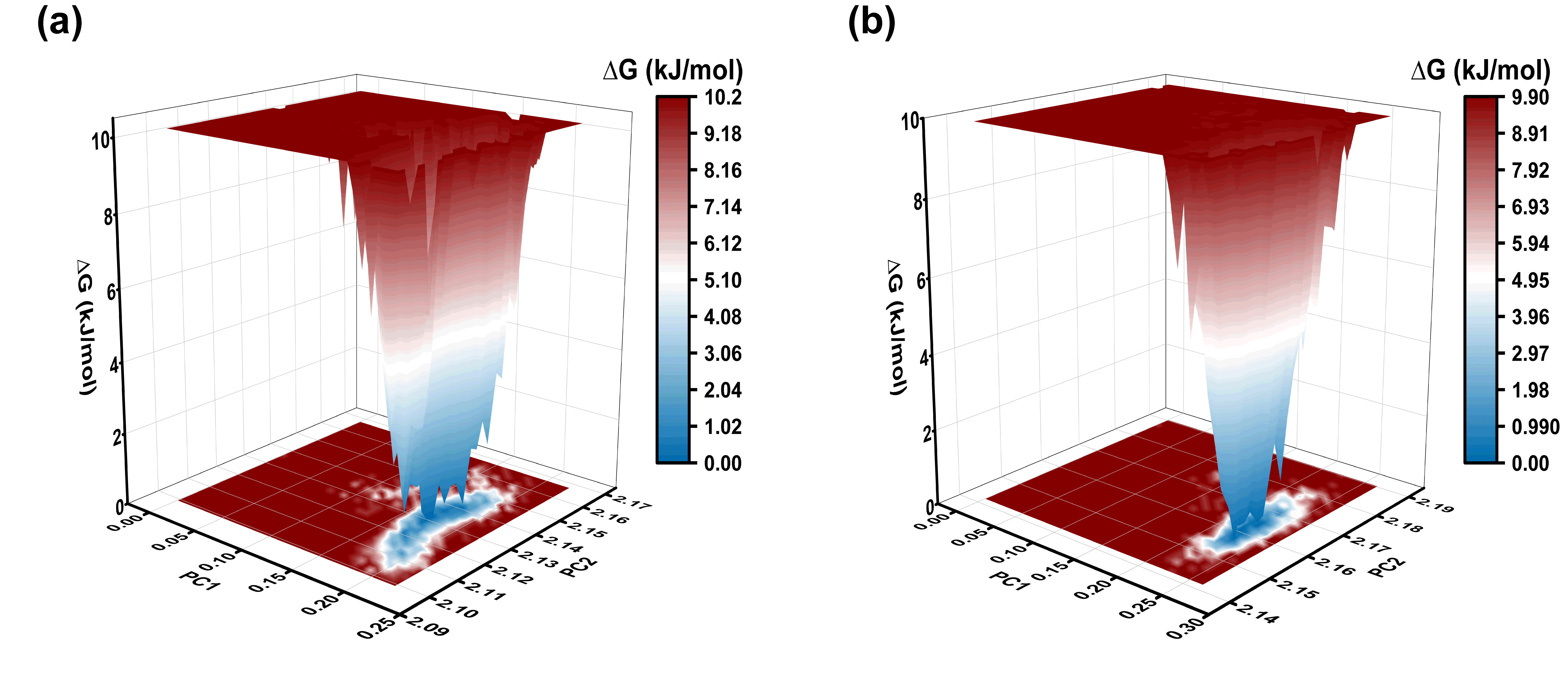


**Figure S6.** Free energy landscape profiles of the substrate entry and product release processes from the 100 ns MD simulations. (a) Substrate entry pathway. (b) Product release pathway.


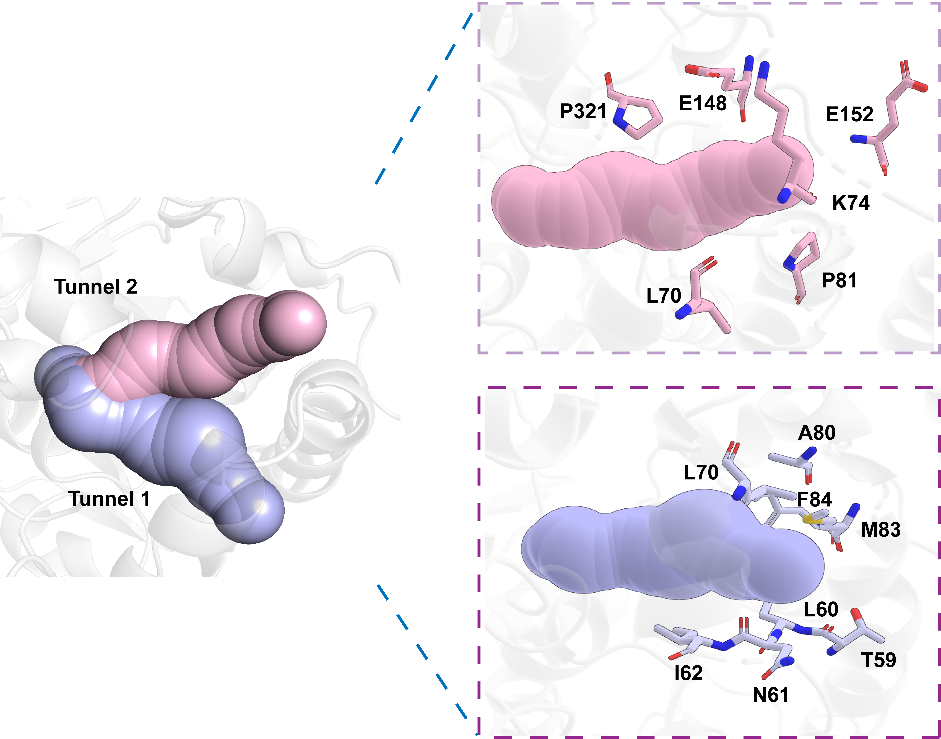


**Figure S7.** Illustration of key amino acid residues in wild-type enzyme, lining tunnel 1 shown as sticks.


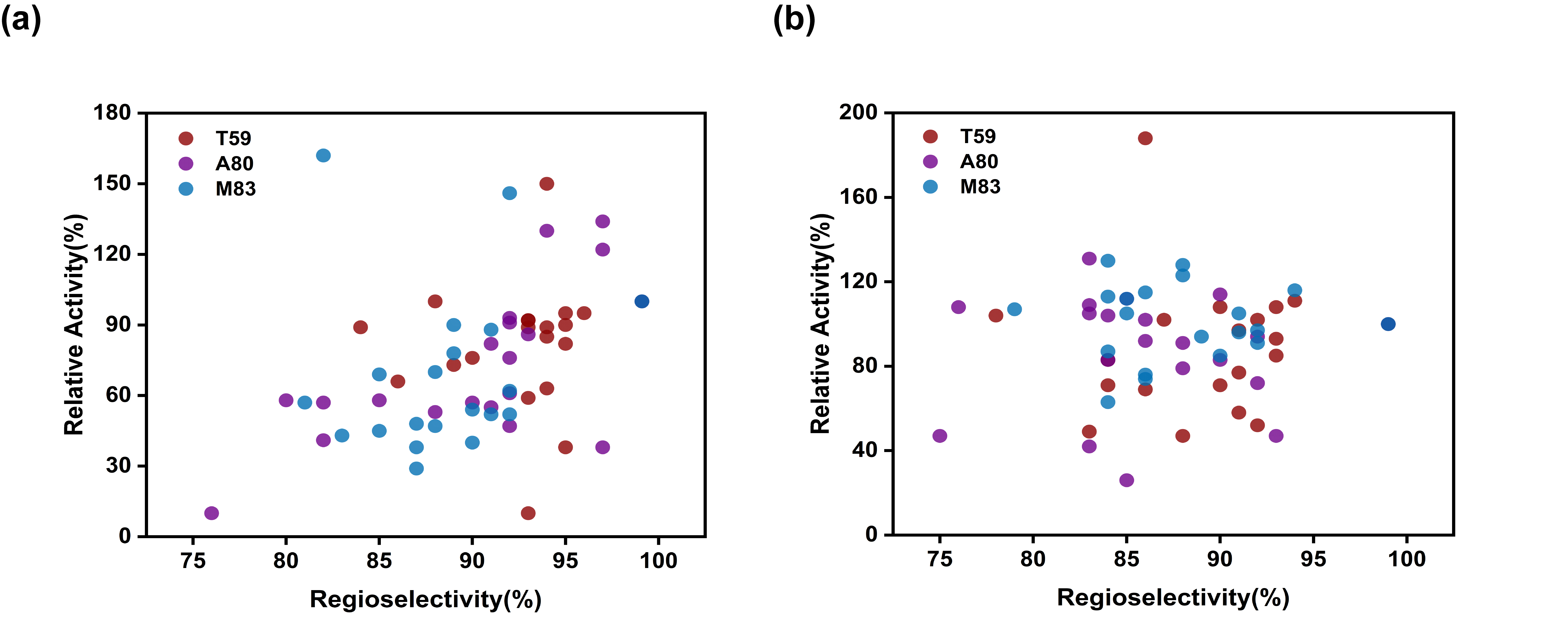


**Figure S8.** Relative activity and regioselectivity of hotspot residues in tunnel 1. (a) Results of saturation mutagenesis of hotspot residues using mutant M1 as template. (b) Results of saturation mutagenesis of hotspot residues using mutant M1-1 as the template.


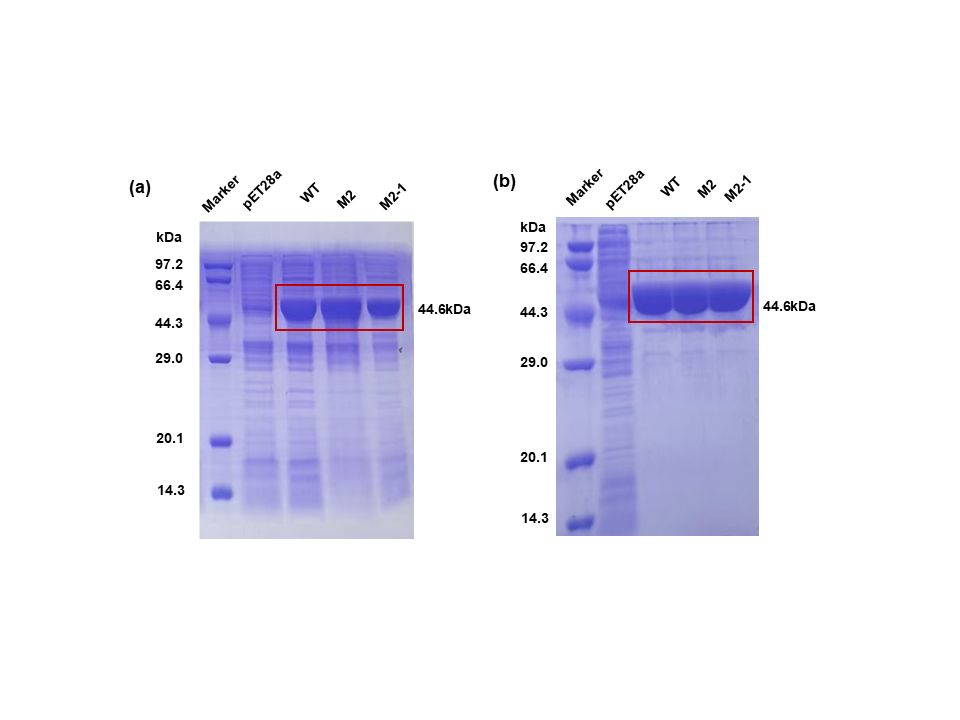


**Figure S9.** SDS-PAGE of the enzyme wild-type, M2 and M2-1. (a) The SDS-PAGE of crude enzyme. (b) The SDS-PAGE of purified enzyme.


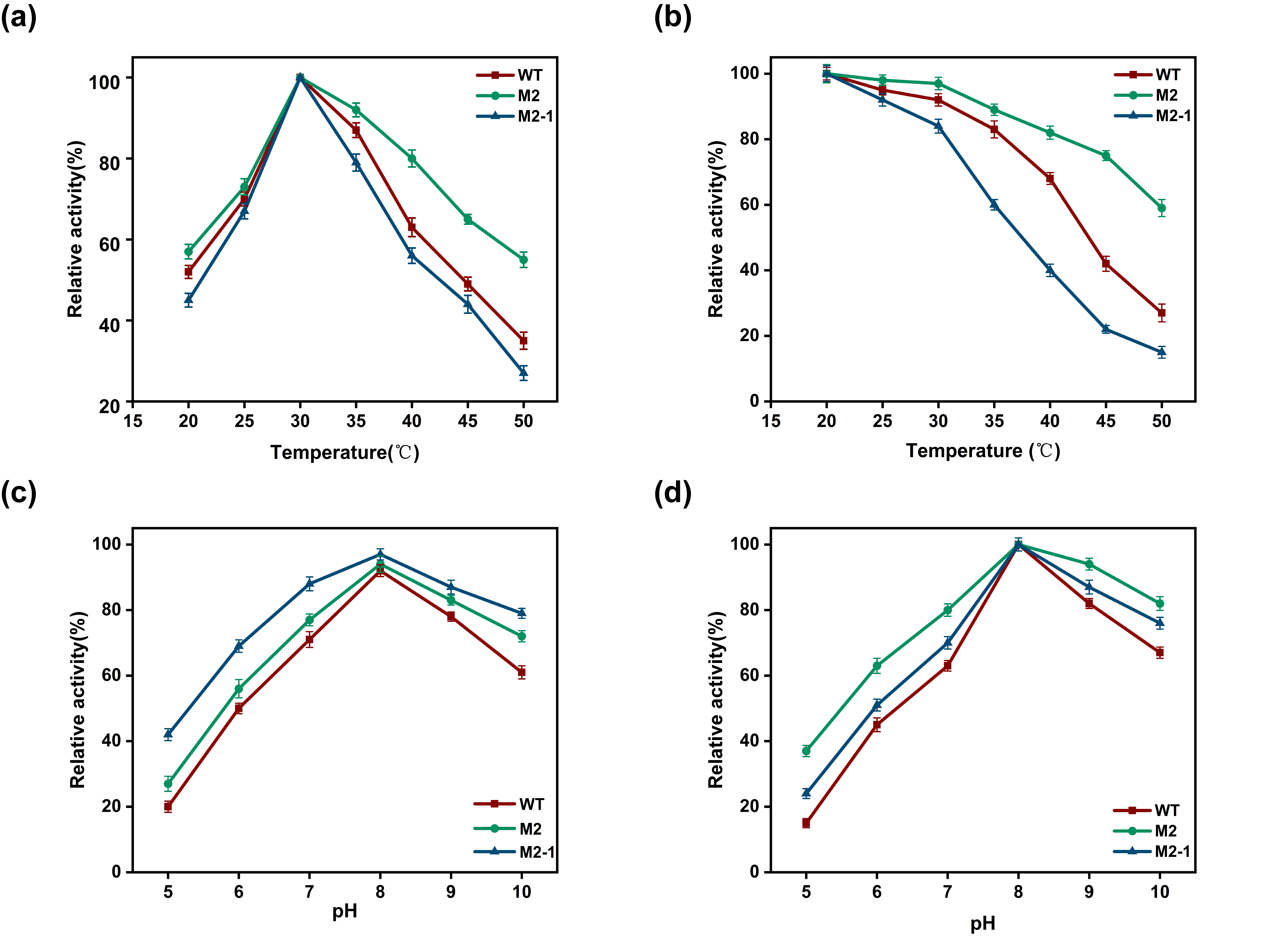


**Figure S10.** Effects of temperature and pH on the enzyme activity of wild-type UGT_BL_1 and its M2 and M2-1 variants. (a) and (b) show the optimal temperature for activity and thermal stability of these enzymes, respectively. (c) and (d) depict the optimal pH for activity and the pH stability range, respectively.


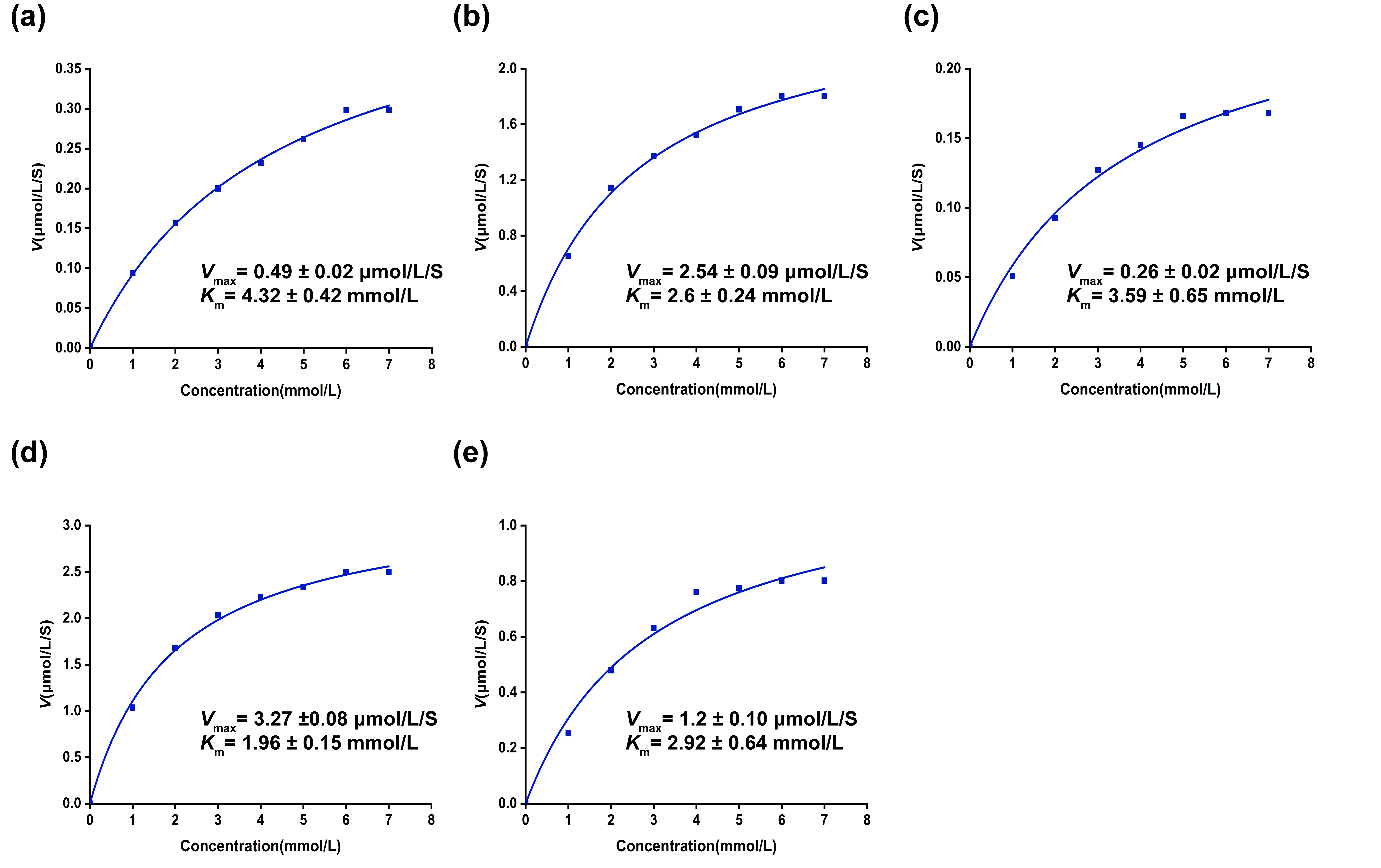


**Figure S11.** Analysis of the enzyme kinetic parameters of wild-type UGT_BL_1 and several mutants towards substrate tyrosol including (a) wild-type UGT_BL_1; (b) M1; (c) M1-1; (d)M2; (e) M2-1, respectively.


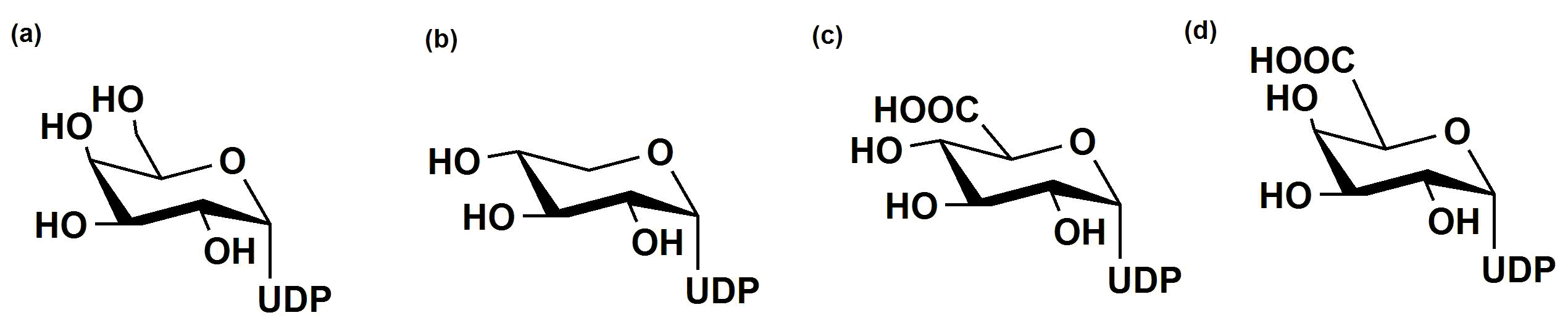


**Figure S12.** Sugar donor substrates used to assess the promiscuity of UGT_BL_1 and its M2 and M2-1 mutants. (a) UDP-α-D-Gal, (b) UDP-α-D-Xyl, (c) UDP-α-D-GlcA, (d) UDP-α-D-GalA.


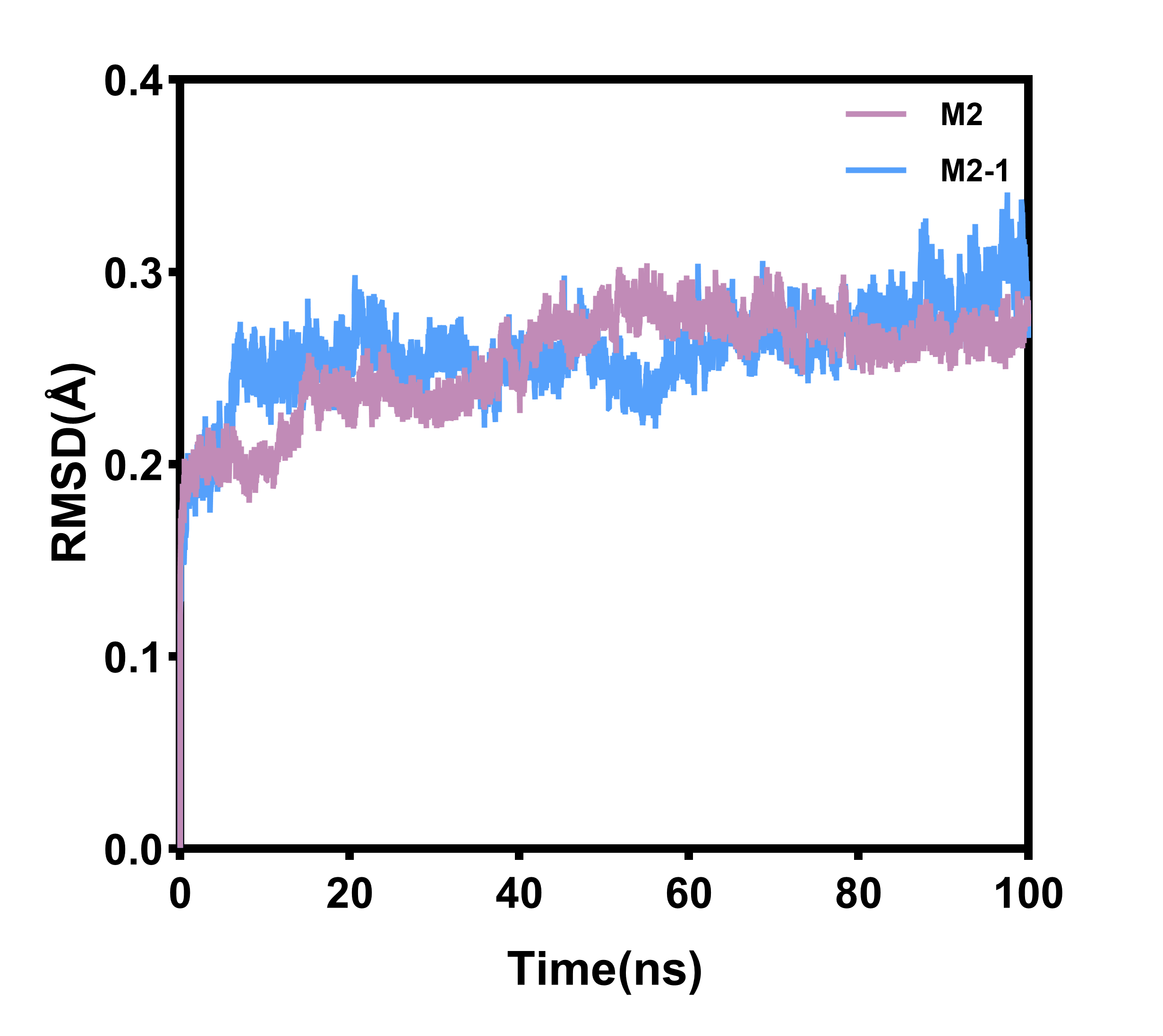


**Figure S13.** Root-mean-square deviation (RMSD) analysis during 100 ns MD simulation s for complexes M2 and M2-1.


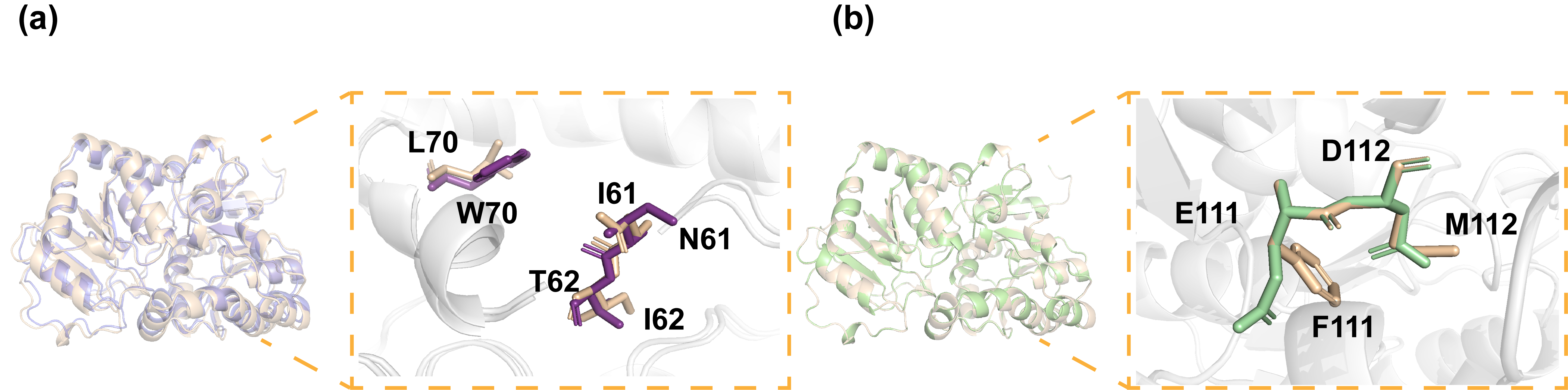


**Figure S14.** Structural superposition between wild-type UGT_BL_1 (wheat colored) and either mutant M2 (purple; a), M2-1(green;b). The mutations are shown as sticks.


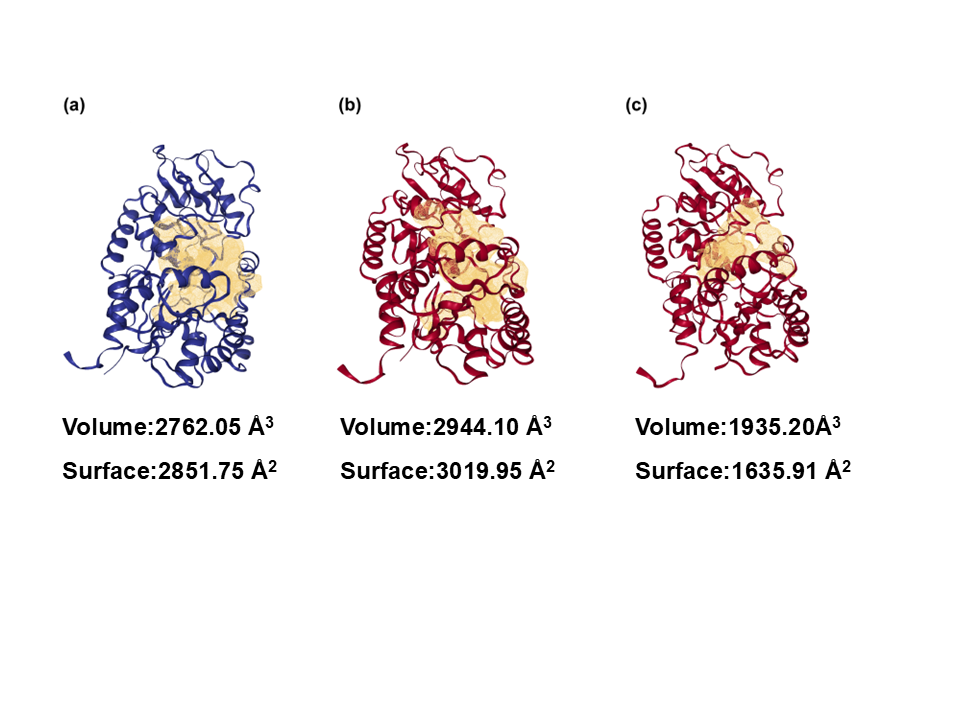


**Figure S15.** Illustration of the shape and volume of substrate binding pocket of UGT_BL_1 (a) and mutants M2 (b) and M2-1 (c).


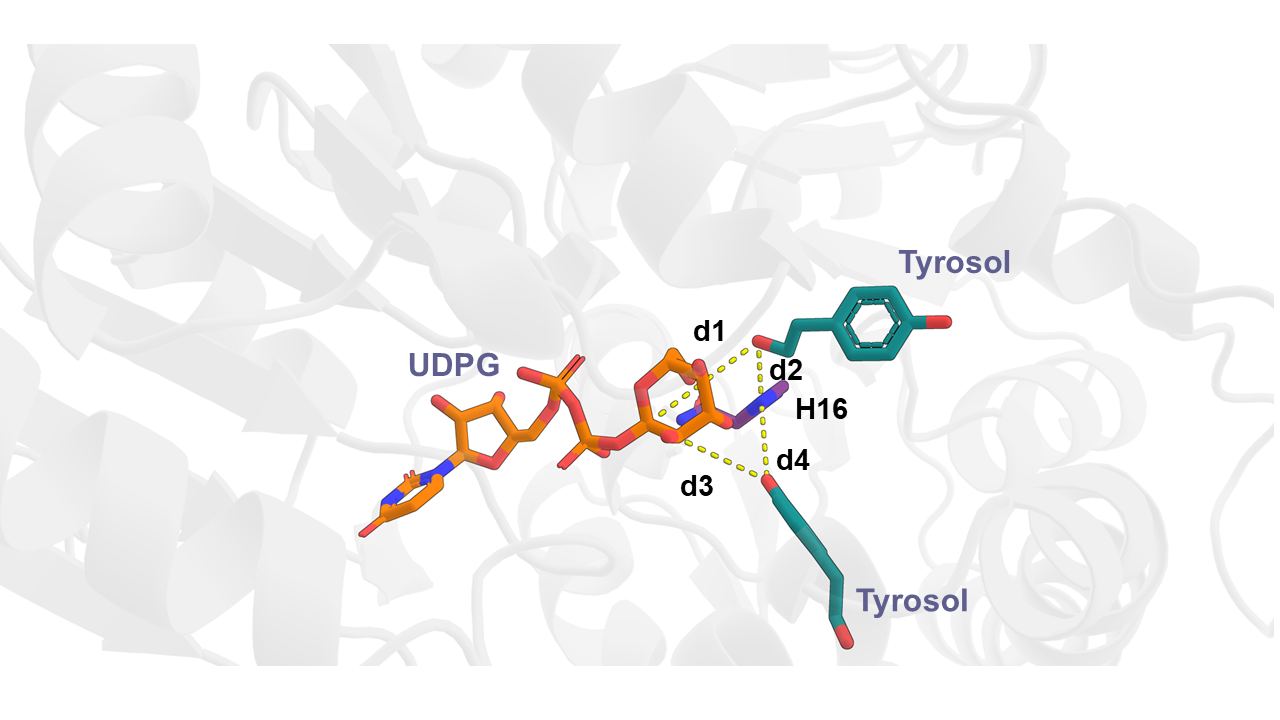


**Figure S16.** Catalytically relevant distances between in the prereaction analysis (d_1_, d_2_, d_3_ and d_4_). Parameter d_1_ represents the distance between C8-OH of tyrosol and Cα of UDPG, while d_2_ represents the distance between C8-OH and the N atom of the catalytic residue H16. The d_3_ represents the distance between C4-OH of tyrosol and Cα of UDPG. The d_4_ represents the distance between C4-OH and N atom of the catalytic residue H16.

**
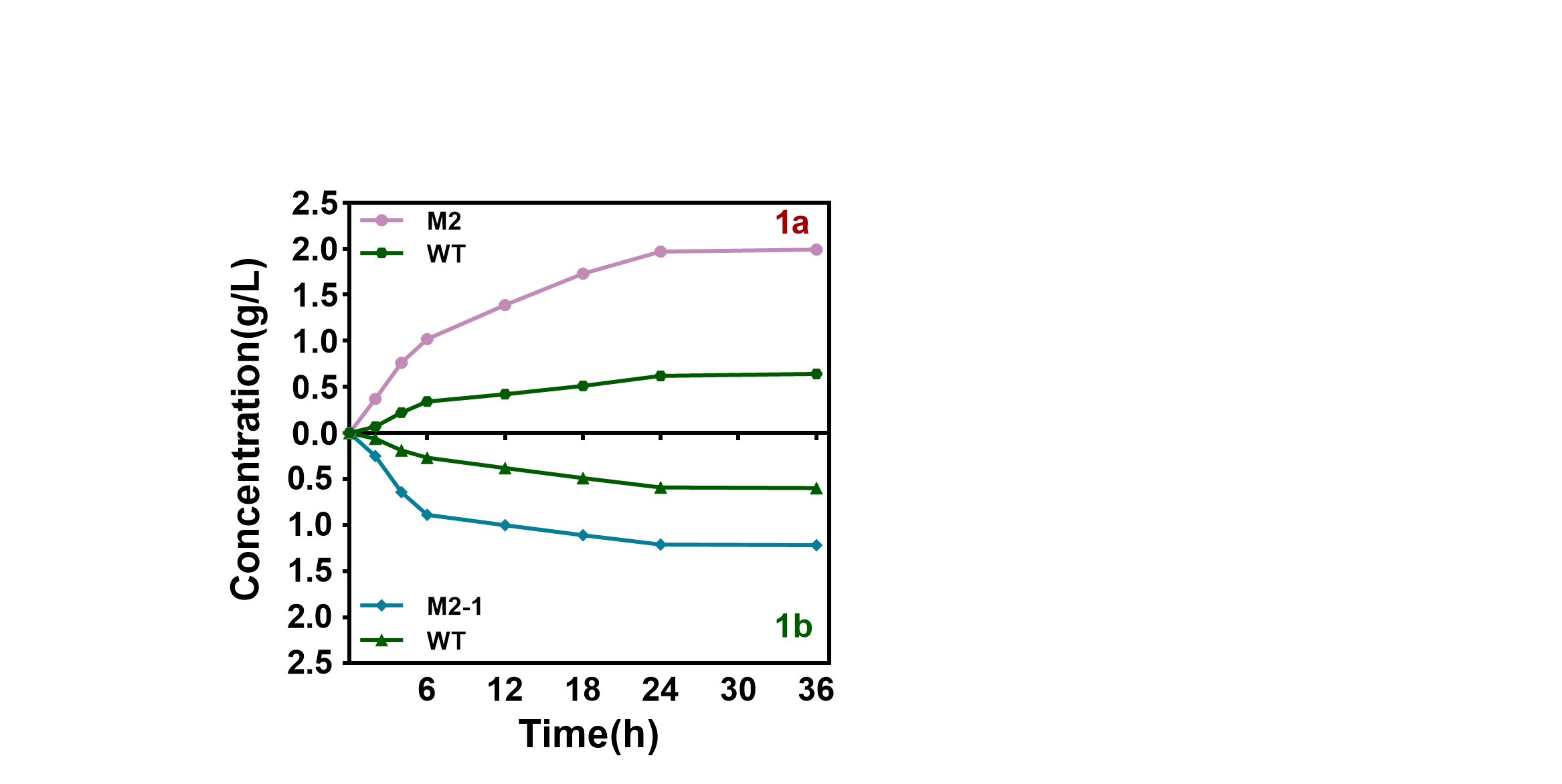
**

**Figure S17.** Biosynthesis of salidroside or icariside D2 using recombinant cells (WT, M2 and M2-1) as biocatalysts.


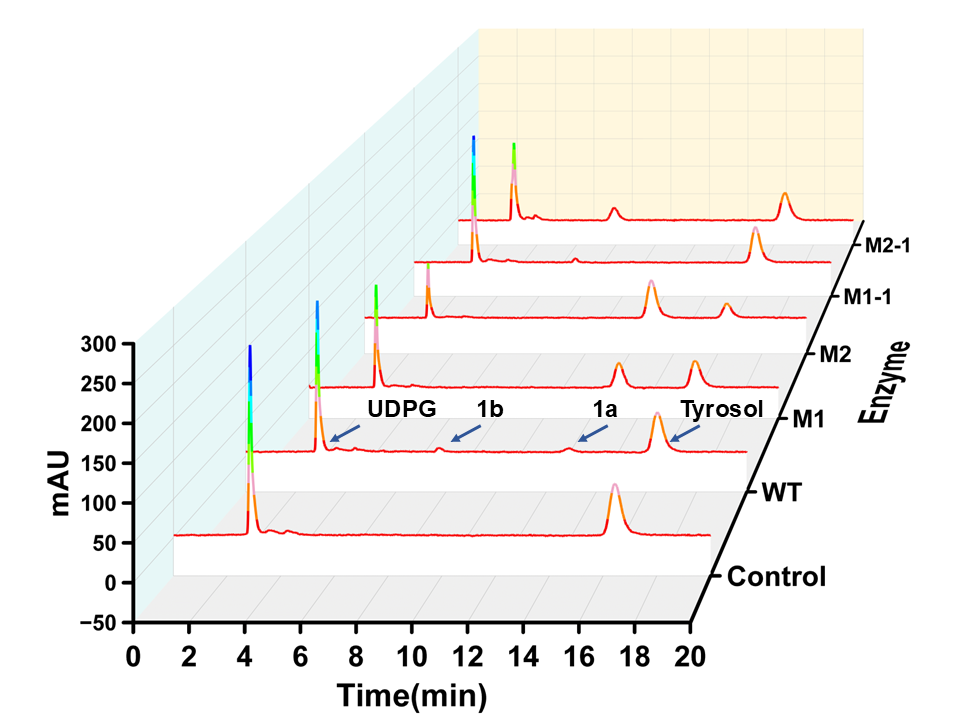


**Figure S18.** HPLC analysis of glycosylation of tyrosol by WT and variants.

**Figure S19.** LC-MS analysis of salidroside catalyzed by mutant M2.

**Figure S20.** LC-MS analysis of icariside D2 catalyzed by mutant M2-1.


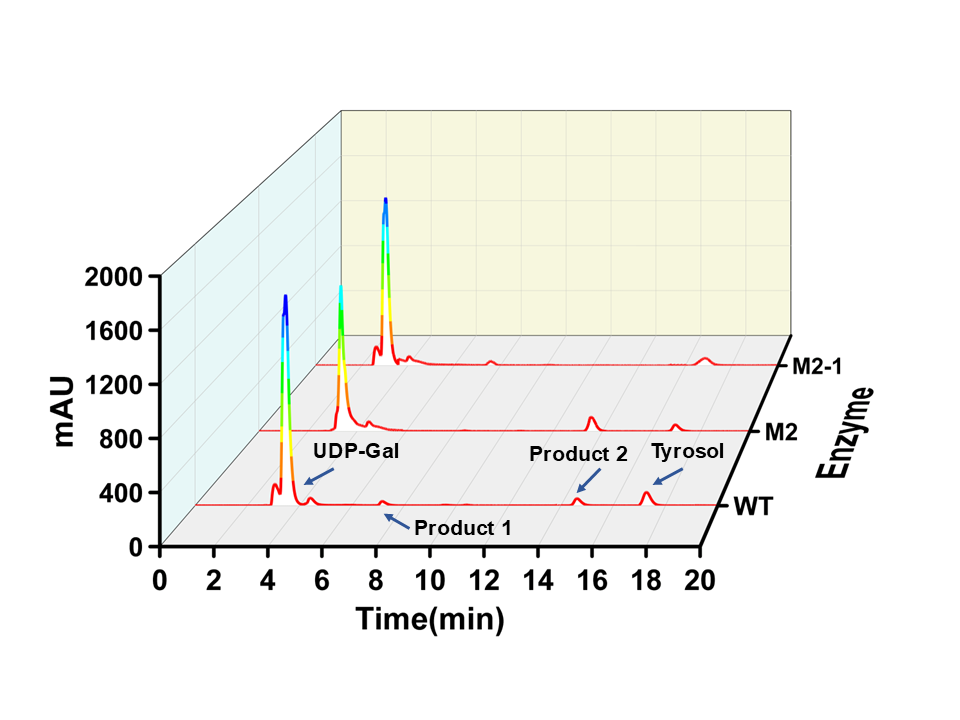


**Figure S21.** HPLC analysis of glycosylation UDP-gal and tyrosol catalyzed by WT and variants.

**Figure S22.** LC-MS analysis of glycosylation UDP-gal and tyrosol catalyzed by WT and variants.


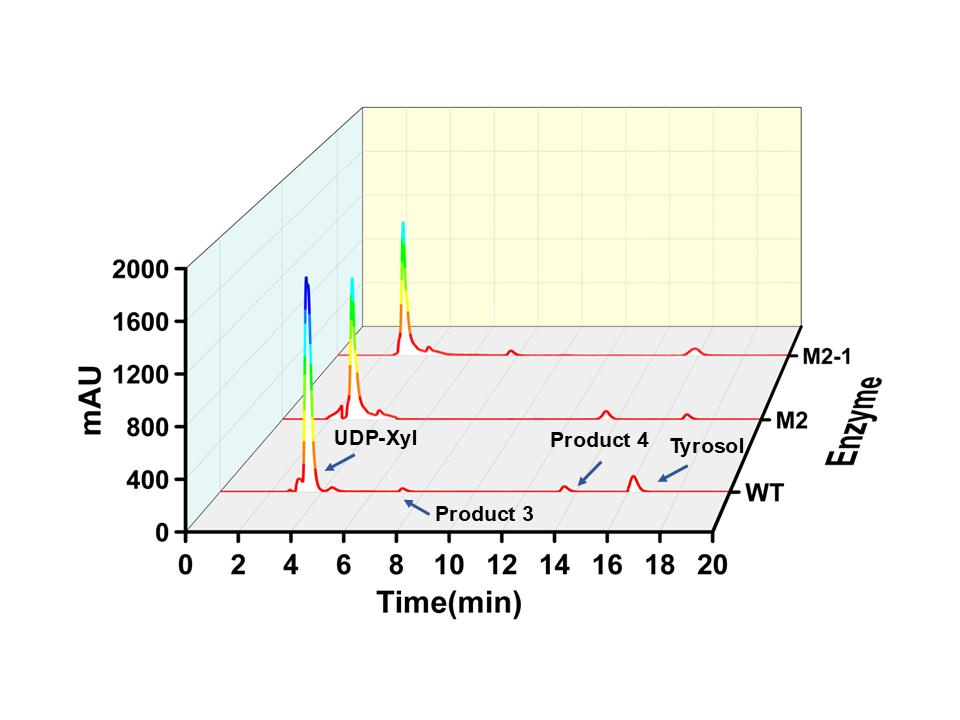


**Figure S23.** HPLC analysis of glycosylation UDP-xyl and tyrosol catalyzed by WT and variants.

**Figure S24.** LC-MS analysis of glycosylation UDP-Xyl and tyrosol catalyzed by WT and variants.


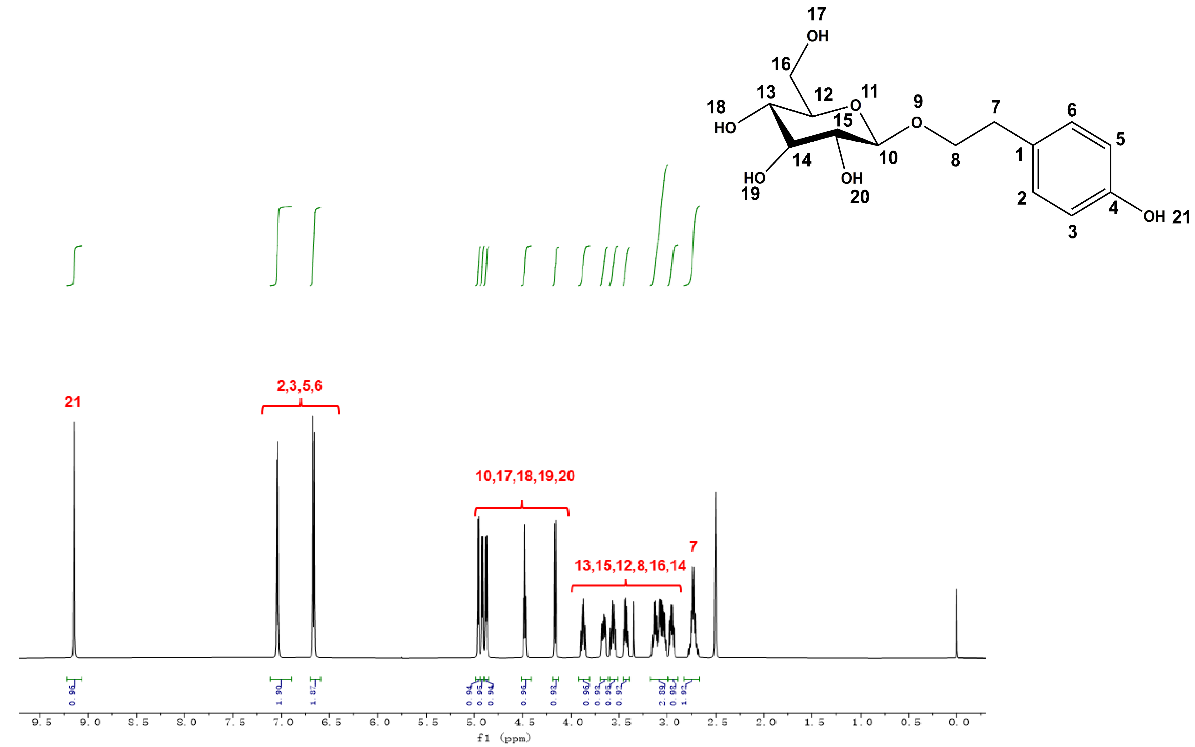


**Figure S25.** ^1^H NMR spectrum of salidroside in dimethyl sulfoxide-d6 (500 MHz).

**
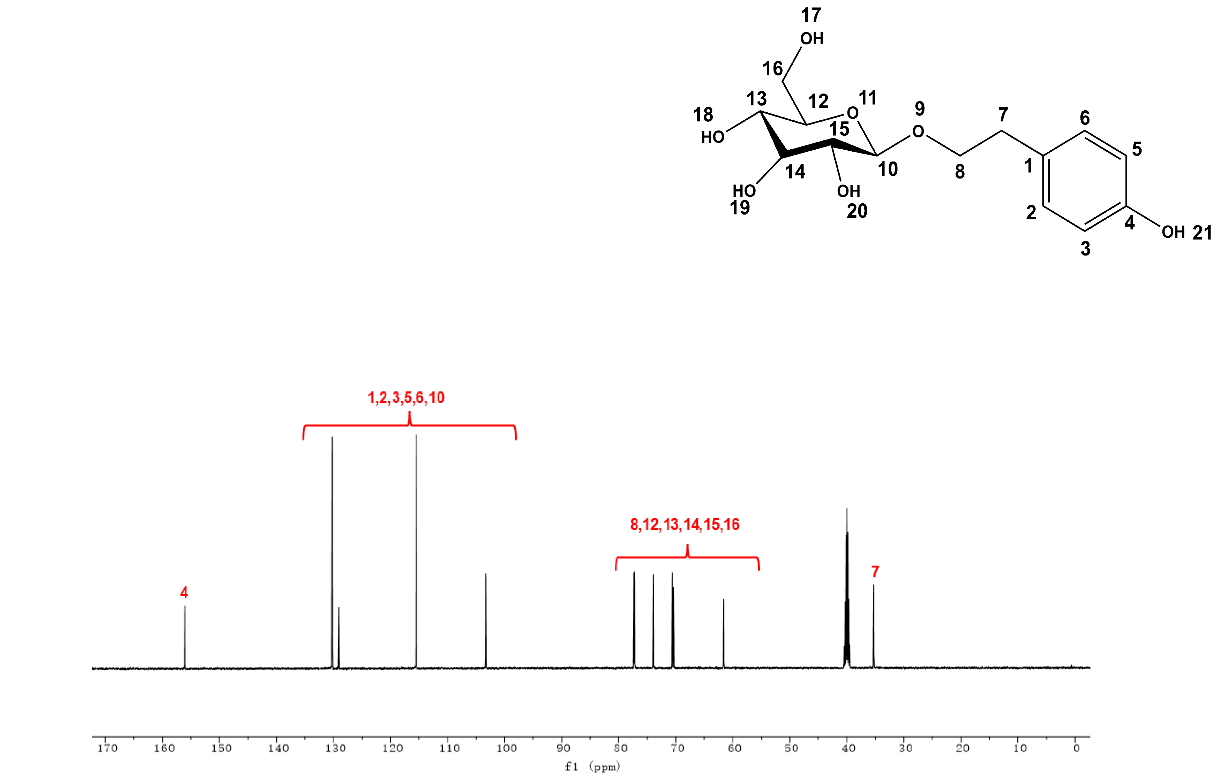
**

**Figure S26.** ^1^C NMR spectrum of salidroside in dimethyl sulfoxide-d6 (125 MHz).


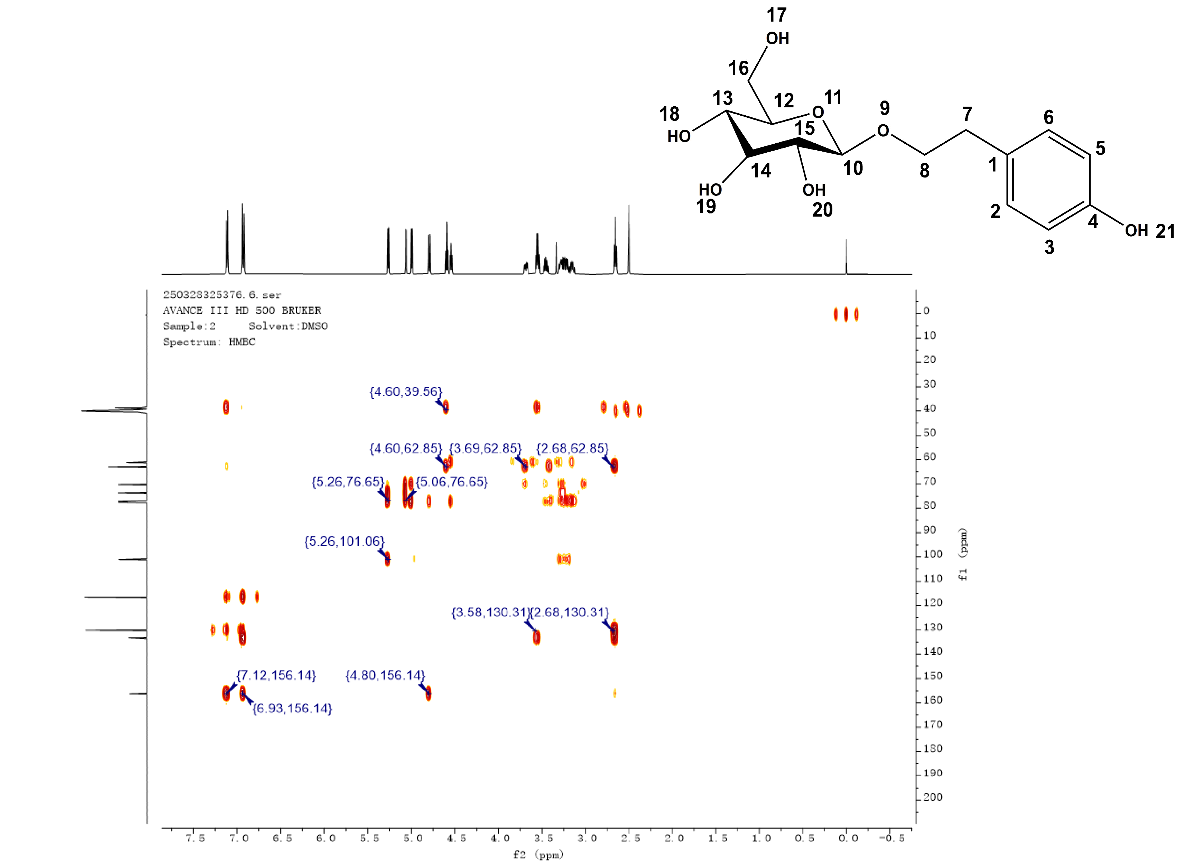


**Figure S27.** HMBC spectrum of salidroside in dimethyl sulfoxide-d6.

**
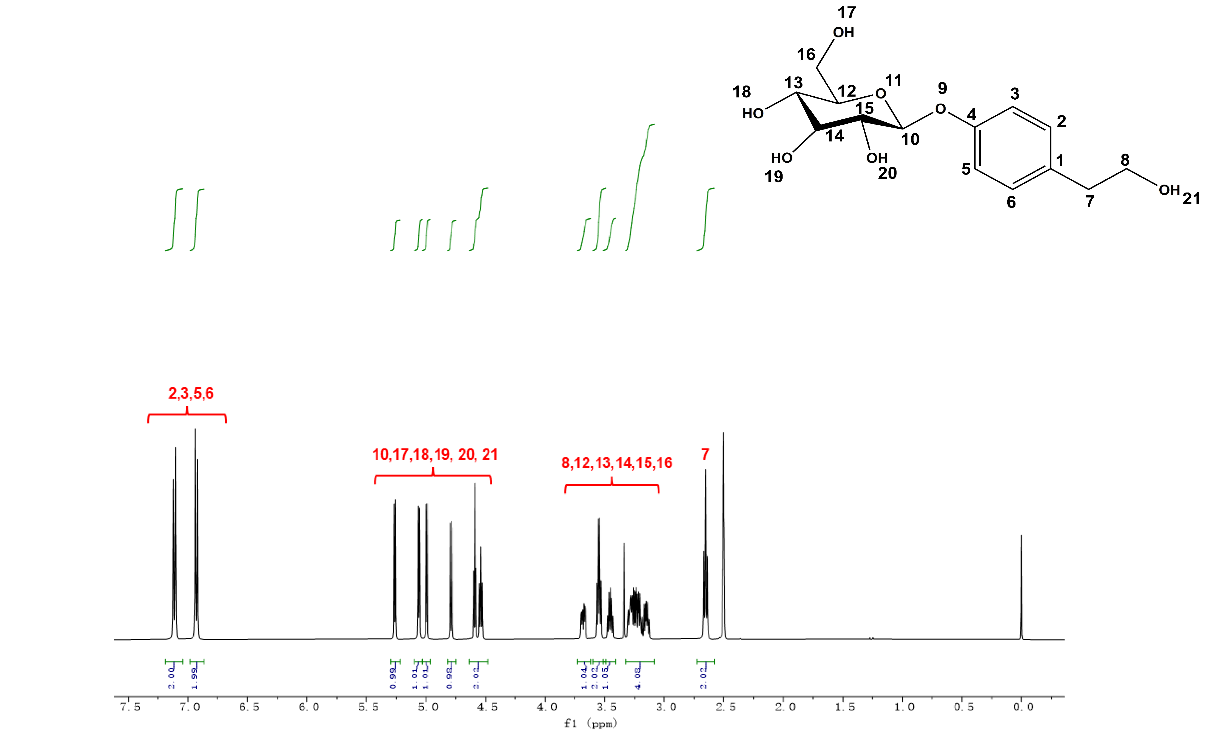
**

**Figure S28.** ^1^H NMR spectrum of icariside D2 in dimethyl sulfoxide-d6 (500 MHz).


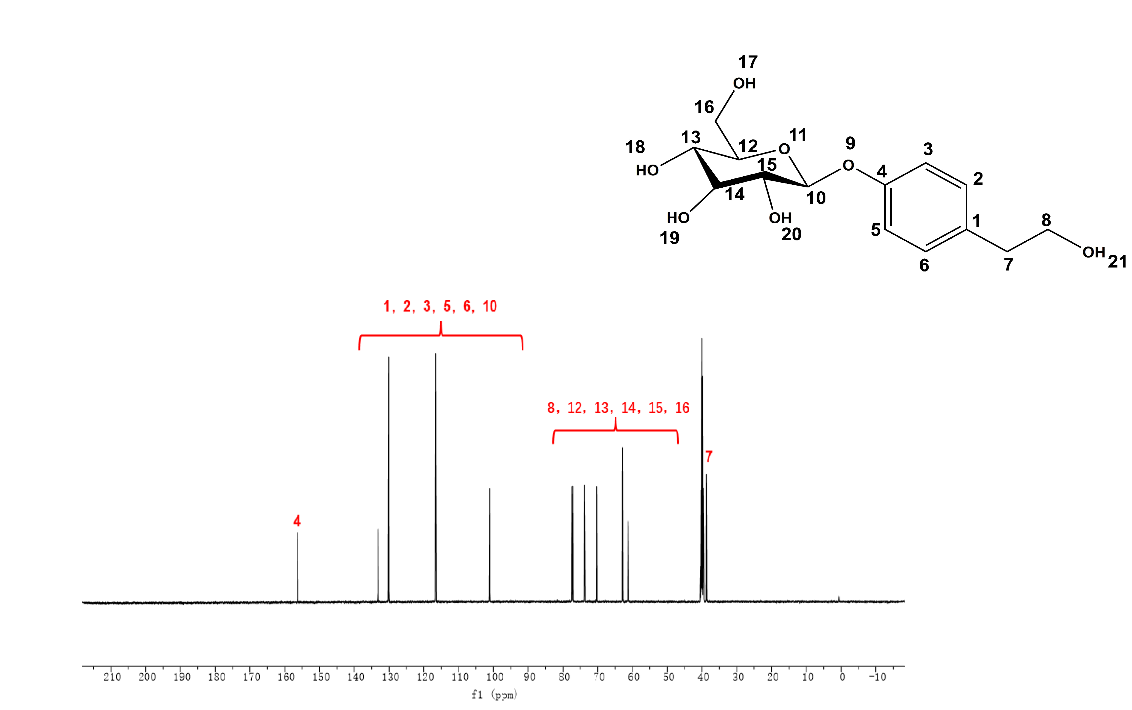


**Figure S29.** ^1^C NMR spectrum of salidroside in dimethyl sulfoxide-d6 (125 MHz).


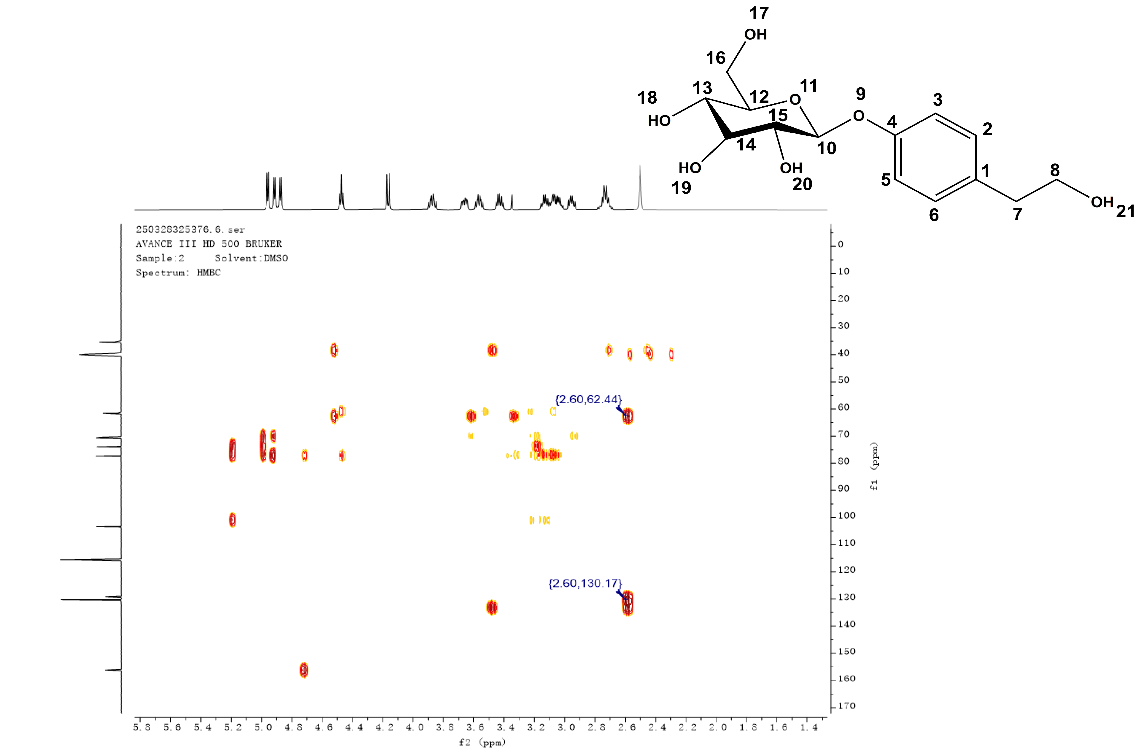


**Figure S30.** HMBC spectrum of salidroside in dimethyl sulfoxide-d6.

**Reference**

[1] Jumper, J.; Evans, R.; Pritzel, A.; Green, T.; Figurnov, M.; Ronneberger, O.; Tunyasuvunakool, K.; Bates, R.; Žídek, A.; Potapenko, A.; Bridgland, A.; Meyer, C.; Kohl, S. A. A.; Ballard, A. J.; Cowie, A.; Romera-Paredes, B.; Nikolov, S.; Jain, R.; Adler, J.; Back, T.; Petersen, S.; Reiman, D.; Clancy, E.; Zielinski, M.; Steinegger, M.; Pacholska, M.; Berghammer, T.; Bodenstein, S.; Silver, D.; Vinyals, O.; Senior, A. W.; Kavukcuoglu, K.; Kohli, P.; Hassabis, D., Highly accurate protein structure prediction with AlphaFold. *Nature* **2021,** *596* (7873), 583-589.

[2] Lindorff-Larsen, K.; Piana, S.; Palmo, K.; Maragakis, P.; Klepeis, J. L.; Dror, R. O.; Shaw, D. E., Improved side-chain torsion potentials for the Amber ff99SB protein force field. *Proteins: Structure, Function, and Bioinformatics* **2010,** *78* (8), 1950-1958.

[3] Giese, T. J.; Panteva, M. T.; Chen, H.; York, D. M., Multipolar Ewald Methods, 1: Theory, Accuracy, and Performance. *Journal of Chemical Theory and Computation* **2015,** *11* (2), 436-450.
